# Flowers under pressure: ins and outs of turgor regulation in development

**DOI:** 10.1093/aob/mcu187

**Published:** 2014-10-06

**Authors:** Léna Beauzamy, Naomi Nakayama, Arezki Boudaoud

**Affiliations:** 1 Reproduction et Développement des Plantes, INRA, CNRS, ENS de Lyon, UCBL Lyon I, 46 Allée d'Italie, 69364 Lyon Cedex 07, France; 2 Laboratoire Joliot-Curie, CNRS, ENS de Lyon, 46 Allée d'Italie, 69364 Lyon Cedex 07, France; 3 Institute of Molecular Plant Sciences, University of Edinburgh, Mayfield Rd, King's Buildings, Edinburgh EH9 3JH, UK

**Keywords:** Flower development, pollen tube, anther dehiscence, flower opening, water potential, osmotic pressure, turgor pressure, hydraulic conductivity, aquaporins, plasmodesmata, osmoregulation

## Abstract

**Background:**

Turgor pressure is an essential feature of plants; however, whereas its physiological importance is unequivocally recognized, its relevance to development is often reduced to a role in cell elongation.

**Scope:**

This review surveys the roles of turgor in development, the molecular mechanisms of turgor regulation and the methods used to measure turgor and related quantities, while also covering the basic concepts associated with water potential and water flow in plants. Three key processes in flower development are then considered more specifically: flower opening, anther dehiscence and pollen tube growth.

**Conclusions:**

Many molecular determinants of turgor and its regulation have been characterized, while a number of methods are now available to quantify water potential, turgor and hydraulic conductivity. Data on flower opening, anther dehiscence and lateral root emergence suggest that turgor needs to be finely tuned during development, both spatially and temporally. It is anticipated that a combination of biological experiments and physical measurements will reinforce the existing data and reveal unexpected roles of turgor in development.

## INTRODUCTION

Plants are made of tiny ‘pressure bombs’. Indeed, walled cells as in plants, fungi or bacteria contain a high hydrostatic pressure termed turgor pressure. Turgor pressure can reach 20 atmospheres, i.e. 2 MPa, a value much higher than the air pressure inside automobile tyres, and plays fundamental roles in structural integrity, morphogenesis and many other aspects of physiological function. Although turgor pressure is often thought to be uniform within a developing organ, it can be variable from cell to cell. It also can change dynamically depending on many intrinsic and extrinsic factors, such as sugar metabolism, changes in the shapes of cells and tissues (including growth) and environmental conditions.

Recent reviews addressed the role of turgor in plant growth (Geitmann and Ortega, 2009; Hamant and Traas, 2010; Robinson *et al*., 2013), hydraulics of plant cells and tissues (Maurel *et al*. 2008; Dumais and Forterre, 2012; Forterre, 2013; Prado and Maurel, 2013; Chaumont and Tyerman, 2014) or the quantification of turgor *in vivo* (Geitmann, 2006; Routier-Kierzkowska and Smith, 2012; Milani *et al*., 2013). Here we try to give a broader view of turgor and its regulation in development. We first summarize the general role of turgor pressure in plant function and then overview the ways by which cells can regulate it. We then review the methods that enable the measurement of water potential, turgor pressure or hydraulic conductivity. Such pieces of information are then placed into specific developmental contexts in three case studies from floral organ differentiation: anther dehiscence, flower opening and pollen tube growth. We finally speculate on the role of the temporal and spatial regulation of turgor in development.

## ROLES OF TURGOR PRESSURE

**Fig. 1.**
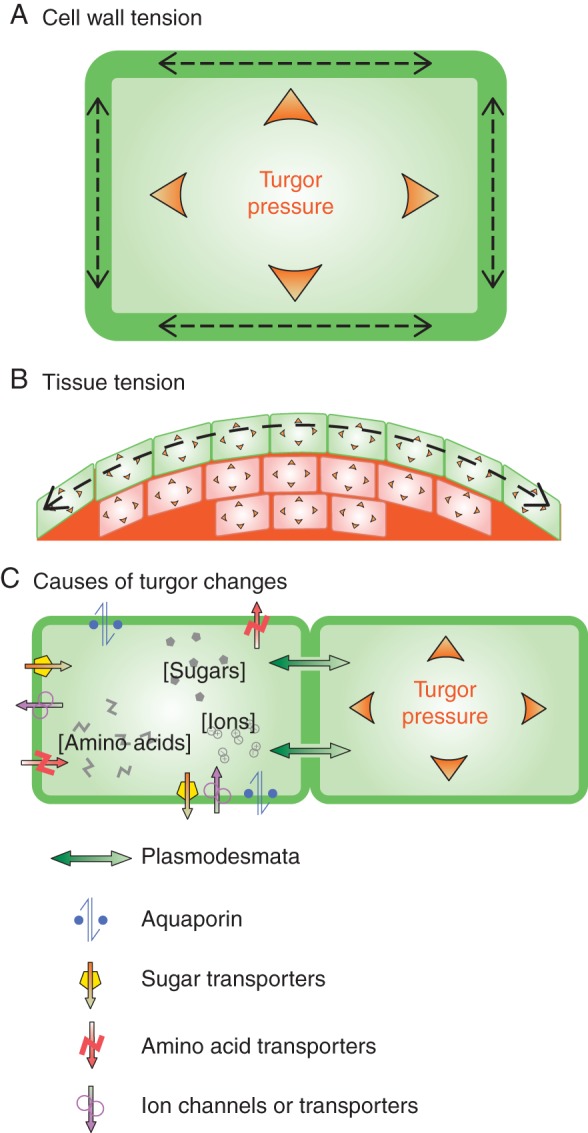
Turgor pressure and its molecular determinants. (A) Turgor pressure puts cell walls in tension. (B) Turgor pressure can also induce tension at the tissue level, for instance in the epidermis. (C) Turgor pressure and water fluxes depend on plasmodesmata, aquaporins, transporters and channels.

Turgor pressure provides structural integrity to each cell and to the tissue as a whole (Fig. 1). At the cellular level, turgor pressure pushes the plasma membrane against the cell wall and causes in-plane mechanical tension within the cell wall (Fig. 1A). The rigid cell wall, which is made of a complex composite of carbohydrate polymers and structural proteins, stretches until it settles at a size and shape where the cell wall can stably withhold the internal pressure. Hence turgor pressure is thought to drive growth (Geitmann and Ortega, 2009; Hamant and Traas, 2010; Robinson *et al*., 2013). The stiffness of a cell comes from both the material properties of the cell wall and the turgor pressure within the cell. In highly turgid cells, the surface stiffness is determined mostly by the turgor pressure and perhaps appropriately is referred to as ‘turgor’ (see Appendix 1). Similarly, at the tissue level, the structural strength of tissues depends on both the cell wall rigidity and turgor pressure in each cell. It can be easily observed that tissues harden when turgor pressure rises and soften and even wilt when turgor falls. Therefore, turgor is essential for the morphology, architecture and engineering soundness of plants. It is also believed that the outermost structure of aerial organs is under tension and withstands the internal pressure (Peters and Tomos, 1996; Hamant and Traas, 2010; Robinson *et al*., 2013); the pressure exerted from the internal cells is borne by the epidermal cells – especially the outermost cell wall on the tissue surface (Fig. 1B).

Given the role of turgor in the structural strength of a plant, it is not surprising that dynamic variations in turgor have been implicated in plant movements (Sibaoka, 1969; Dumais and Forterre, 2012; Forterre, 2013), for instance in the circadian movement of leaves. The most studied example is stomata, which are open only when turgor is high enough in guard cells.

In addition to the structural contributions, turgor pressure also affects physical conditions within the cell and subsequently cellular function and biochemistry. It presses the plasma membrane against the cell wall, possibly thinning the membrane as a result (Coster, 1976). An increase in the cellular hydraulic pressure is often associated with an increase in cytoplasmic concentration so that cytoplasmic crowding modifies the macromolecular conformation and interactions (van den Berg *et al*., 2000; Ellis, 2001; Briegel *et al*., 2014). Intracellular membrane dynamics, as well as organelle sizes and shapes, also seem to be sensitive to turgor pressure; for example, the higher the turgor pressure, the higher the energetic barrier for endocytosis to occur (Fricke *et al*., 2000).

Furthermore, changes in turgor pressure could be involved in signal transduction pathways. Environmental conditions impact turgor pressure; high salinity or water stress reduce turgor, whereas hypo-osmotic conditions (as in flooding) and compression due to stomping by animals or bending by wind, for example, would increase the pressure. Biotic stress, for instance wounding caused by microbe infection, would release turgor pressure. Thus some of the signal transduction and molecular pathways activated in response to environmental stresses could be downstream of cues associated with changes in turgor pressure or other mechanical alterations (Walley *et al*., 2007). In fact, osmotic conditions of the cell are so interlinked with turgor pressure that it is often thought that cells measure their intracellular pressure via osmosensing (Urao *et al*., 1999; Kumar *et al*., 2013). Alternatively, cells are thought to sense turgor pressure from their expansion or shrinkage following changes in the internal pressure. Mechanosensitive proteins embedded in the plasma membrane which become activated when cells are deformed [for example those reviewed in Monshausen and Haswell (2013), e.g. stretch-activated channels] can indirectly trigger responses to turgor changes. It is likely that there are many different ways by which cells sense physical parameters such as turgor pressure; just about any of the diverse ways turgor pressure can affect cells, as described above, could be an input to induce cellular responses. In this framework, turgor would be at the same time an integrator of environmental and developmental cues and an entry point for various signalling pathways.

In addition to the cellular physiological functions, turgor pressure and its gradient within a tissue can provide positional information, driving water and molecular movement directionally. In 1930, Ernst Münch proposed the so-called ‘pressure or mass flow hypothesis’ to explain how sugars are transported from sink to source in plants (Taiz and Zeiger, 2010). The phloem cells at source have a high concentration of sugar that draws water into them, creating a gradient in turgor pressure, which is higher in the source and lower in the sink. Movement of the phloem sap occurs by bulk (mass) flow down the turgor gradient to the sink. For nearly a century, it has been the most dominant theory for the mechanism driving long-distance transport. An even longer standing theory (the cohesion–tension theory proposed by Joly and Dixon in 1894) dictates that the movement of water and nutrients through the xylem also follows the gradient in water potential. Unlike in the phloem, however, xylem transport occurs down the pressure gradient from the root to the leaf, where a negative pressure is generated by water evaporation into the air spaces in the mesophyll.

## REGULATION OF TURGOR PRESSURE

Turgor pressure increases or decreases when the water content changes within the cell (Fig. 1C). In an ideal equilibrium situation with no active regulation of water content and no water flow through the plant, turgor pressure would only be determined by differences in osmotic potential between compartments (Appendix 1), e.g. between a cell and its extracellular space. Out of equilibrium, water influx or efflux can be driven directly or indirectly. Random water movement across the membrane does happen at a low rate; however, water molecules are transferred into and out of a cell much more efficiently through openings in the cell boundary (Haines, 1994). The direct water movement occurs through such ‘holes’ – water channels embedded in the plasma membrane, known as aquaporins, and the intracellular cytoplasmic connections called plasmodesmata. Water passes through these openings following gradients in water potential (see below). Hence water movement can be facilitated indirectly by changing the differential osmotic potentials across the cell boundary. Alternatively, if water flow is driven, for instance by evaporation, an increase in the difference in water potential between compartments occurs (Appendix 1), that depends on the conductivity of openings between the two compartments, such as plasmodesmata or aquaporins. Therefore, closure of plasmodesmata or gating of aquaporins can contribute to the regulation of turgor.

### Aquaporins

Water channels are membrane intrinsic proteins of a large family (e.g. 35 have been identified in the genome of *Arabidopsis thaliana*; see Johanson *et al*., 2001). Dubbed aquaporins, they are diverse yet universal and found in all living kingdoms. Aquaporins are localized to various membranes within a cell – mostly the plasma membrane or vacuolar membrane (also known as the tonoplast), but also in other compartments (Chaumont and Tyerman, 2014). Plasma membrane-localized aquaporins are called plasma membrane intrinsic proteins (PIPs), and they can be classified into two types, PIP1 and PIP2, based on protein sequence similarities.

PIPs are small (24–34 kDa) proteins with six transmembrane domains, which are arranged in a ring-like conformation. Water molecules are transported inside the ring by osmosis, in both the influx and efflux directions, depending on the difference between the cytosolic and apoplastic water potentials (Murata *et al*., 2000). They usually form tetramers, but plant aquaporins seem to have diversified more extensively, and some of them make up heteromers (Fetter *et al*. 2004). This variety may be reflected in their functional specificities; some of them are water-specific channels but some others are also permeable to other molecules, such as glycerol, CO_2_, boron and small organic solutes (Henzler and Steudle, 2000; Chaumont *et al*., 2005).

Aquaporins seem to mediate most of the water transfer across the plasma membrane. When the water permeability of the plasma membrane was measured in isolated protoplasts, it was found to be highly sensitive to mercury, a potent inhibitor of aquaporin function (Ramahaleo *et al*., 1999). The measurement varied significantly (nearly a thousand-fold) depending on the species, tissue types and developmental stages, suggesting that water movement is tightly regulated during development. In peach trees, the end of the dormancy correlates with higher expression of tonoplast and plasma membrane aquaporins and an increase in the water content in the bud (Yooyongwech *et al*., 2008). Overexpression of an aquaporin gene (*AtPIP1;2*) produced an increase in the growth rate, transpiration rate and photosynthetic efficiency in *Nicotiana tabacum* (Aharon *et al*., 2003), though these effects might also be ascribed to the deregulation of stomata.

The water channels are not constitutively open, and their function can be gated, for instance through post-translational modifications (Chaumont *et al*., 2005). Their closure or opening are generally associated with environmental changes and stresses that plants experience. For instance, temperature affects aquaporin function. Cold treatments reduced turgor, hydraulic conductivity and active nutrient transport in roots of *Cucumis sativus* (Lee *et al*., 2004). Heavy metals (e.g. mercury, gold and silver) also close the water channels (Zhang and Tyerman, 1999; Niemietz and Tyerman, 2002). The dark-induced increase in leaf hydraulic conductivity in *A. thaliana* depends on the phosphorylation of aquaporins (Prado *et al*., 2013). Furthermore, cytosolic acidification upon anoxia and other stresses inhibits water uptake via aquaporin (Tournaire-Roux *et al*., 2003).

Factors known to be involved in stress-induced signal transductions, such as reactive oxygen species (ROS; e.g. hydrogen peroxide), also affect aquaporin activity (Henzler and Steudle, 2000; Boursiac *et al*., 2008). Treatments with divalent cations (especially Ca^2+^) or H^+^ inhibited water transport in suspension cultured cells, indicating that external acidity and Ca^2+^ can also influence the gating mechanisms (Gerbeau *et al*., 2002). In addition, aquaporin function is regulated via direct phosphorylation by membrane-associated calcium-dependent protein kinases (Johnson and Chrispeels, 1992; Chaumont *et al*., 2005).

### Plasmodesmata

Plasmodesmata are symplasmic connections between two adjacent cells. They are the tunnels across the cell wall through which water and solutes of all kinds (e.g. nutrients, hormones, RNAs and proteins) can travel, as long as they are smaller than the size exclusion limit (Lucas and Lee, 2004; Burch-Smith and Zambryski, 2012). Plasmodesmata are indispensable for cell–cell communication among neighbouring cells, as well as for long-distance transport and signalling. They are also important for intercellular exchange of water and osmo-active solutes, and thus for the equilibration of turgor pressure. Based on the observations from fluorescent recovery after photobleaching, the speed of movement of small molecules through plasmodesmata was calculated to be around 2·5–4·1 μm s^–1^ for a single epidermal cell wall in the root basal meristem (Rutschow *et al*., 2011). In other words, molecules can move from one cell to another almost instantaneously.

Plasmodesmata are complex structures that gather in cytoskeletons and endomembranes (especially specialized endoplasmic reticulum membranes). Like aquaporins, they are not passive ‘holes’ and their permeability can be gated, although it is still unclear if the gating can be complete and thus can block water and small molecules. Plasmodesmata exhibit sophisticated selectivity in macromolecule trafficking that depends on the size and species of mRNAs and proteins. The size exclusion limit measured using florescent-labelled dextran was found to be around 700–800 Da in the mesophyll cells of tobacco leaf (Wolf *et al*., 1989). This limit was increased by >10-fold when the leaf was infected with *Tobacco mosaic virus*, probably reflecting a strategy of the virus to spread through the plant body efficiently.

Callose deposition to the apoplastic region surrounding plasmodesmata seems to be the key event leading to the closure of plasmodesmata and prevention of protein traffic within (Vaten *et al*., 2011; Li *et al*., 2012). This callose deposition can be induced by ROS accumulation due to stress (Benitez-Alfonso *et al*., 2011). Alternatively, a cytoskeleton-mediated mechanism could act in plasmodesmata gating. A surge of cytoplasmic free Ca^2+^ induces a rapid closure (within 5 s) of plasmodesmata in *Zea mays* suspension cultured cells (Holdaway-Clarke *et al*., 2000). Since this process is too fast to be due to callose deposition or reabsorption, it is more likely to be due to actin, myosin and/or centrin action, as those proteins are localized at the plasmodesmata in higher plants. Turgor pressure, especially a sharp difference in pressure between adjacent cells, has been shown to regulate protein trafficking through plasmodesmata. The protein movement was blocked when the turgor pressure dropped suddenly in one cell by 200 kPa but not in the other cells of the multicellular trichomes in *Nicotiana clevelandii* (Oparka and Prior, 1992).

Plasmodesmata, with their selective open/closed states, can create separate multicellular symplasmic domains within a tissue, where the cells may establish distinct molecular make-ups. For example, in the birch shoot apical meristem, tunica layers are separated from the corpus, and the central zone and peripheral zone consist of separate symplasmic domains (Rinne and van der Schoot, 1998). The tissues surrounding gametophytes also demarcate distinct symplasmic domains (Imlau *et al*., 1999). Flowering in arabidopsis seems to correlate with symplastic isolation of the shoot apex from the phloem (Gisel *et al*., 1999). A drastic reduction in fluorescent tracer transport, which travels from vasculature tissue to the shoot apex, was observed when plants were submitted to flowering-inducing long day conditions. Meanwhile, in short day conditions, water transport stayed high. The long day treatment not only promoted flowering but also inhibited the movement of small molecules (<520 Da) into the shoot apex (Gisel *et al*., 1999).

Specific closure of plasmodesmata is important for key developmental events, because of the water movement and/or diffusion of other molecules. The beginning of the rapid elongation phase of *Gossypium hirsutum* fibre is accompanied by the closure of plasmodesmata, which might enable a higher pressure to be maintained in the fast growing fibre cells and drive rapid cell elongation (Ruan *et al*., 2001). During flower development, LEAFY and DEFICIENS, transcription factors that specify floral meristem identity in arabidopsis and floral organ identity in *Antirrhinum majus*, respectively, have been shown to move symplastically through plasmodesmata between the epidermal and internal cell layers (Perbal *et al*., 1996; Sessions *et al*., 2000). Whether concomitant water movement is important or not, to facilitate the protein translocation or for any other reasons, has not been directly tested yet.

### Osmoregulation

Osmoregulation is the osmotic adjustment of the cell to control its water content by increasing or decreasing the cytosolic and vacuolar concentrations of osmotically active molecules. It should be noted that even though sub-cellular osmoregulation takes place, the osmotic pressure in the vacuole approximately equals that of the cytoplasm at equilibrium because the tonoplast (unlike the cell wall) cannot sustain significant differences in pressure. Therefore, the function of vacuolar compartmentalization is not for generating differences in pressure, but probably for removing solutes that should not be present in the cytoplasm at high concentration. Plants' remarkable capability to osmoregulate has been well documented and researched for their response to water deficits or high salinity (Morgan, 1984). However, osmoregulation also occurs almost constitutively in healthy plants. During a photoperiod, turgor adjusts to the periodic fluctuations in sugar concentration and water conductance (Haydon *et al*., 2011). In addition, in order to maintain turgor during growth, cells would need to osmoregulate.

The three major classes of osmo-active molecules modulated in osmoregulation are ions, sugars and amino acids (Fig. 1C). The molecular mechanisms to adjust the concentration of each type of the osmolytes are as follows.

#### Ions

The ion concentrations in the cytosol and the organelles depend on the balance between the influx and efflux across the plasma and intracellular membranes. The transport takes place down the electrochemical potential (i.e. Nernst potential) for channels. This is the case for some sym-/antiporters, which pump ions in or out using the concentration gradient of another ion (often H^+^). Some other ion transporters (e.g. H^+^-ATPase) require ATP and energy input.

Osmoregulation via ions predominantly occurs through K^+^ and its counterions. Na^+^/H^+^ antiporters NHX1 and NHX2, which pump H^+^ out of and Na^+^ and/or K^+^ into the vacuole, are necessary for the homeostasis of vacuolar pH and K^+^ accumulation. These antiporters can act to sequester the osmo-active cations in the vacuole, thus being able to drive water movement into the vacuole first and ultimately to the whole cell, increasing the turgor pressure. When these genes were impaired, the vacuole became more acidic and 70 % less enriched in K^+^, resulting in a dwarf phenotype with reduced cell expansion (Bassil *et al*., 2011). On the other hand, *NHX1* overexpression conferred salt tolerance (Apse *et al*., 1999).

#### Carbohydrates

Sugar production or breakdown can also elevate the osmotic potential of the cell. Carbohydrate metabolism, especially photosynthetic production of sucrose, is important for cell division and expansion. It is also important for photoperiodic turgor control and phloem transport of sugars. In sink tissues, storage polysaccharides break down to increase the osmotic potential; for example, starch splits into glucose, fructose and/or malic acid, increasing the concentration of osmo-active solutes (Taiz and Zeiger, 2010).

The sucrose concentration can increase in the cytosol because of sucrose transport. Sucrose transporters, such as sucrose transporter 1 (SUT1), are monosaccharide transporters that belong to a single gene family. They are high-affinity sucrose–proton symporters, which transport sucrose and protons in the same direction across a membrane. SUT1 in *Solanum lycopersicum* and *S. tuberosum* localizes to the phloem sieve elements, where the phloem loading takes place (Lalonde *et al*., 1999). *SUT1* expression changes in response to sugar availability, and it is upregulated in the sink tissues. Reduced SUT1 levels result in a 5- to 10-fold increase in carbohydrate concentration in the leaves (Riesmeier *et al*., 1994).

#### Amino acids

Amino acids are osmo-active, and their concentrations are modulated during osmoregulation. Proline in particular is targeted in osmoregulation. Proline is a proteinogenic amino acid with an exceptional conformational rigidity. It has been thought to be an inert, compatible osmolyte that protects sub-cellular structures and macromolecules under osmotic stress (Szabados and Savouré, 2010). The proline concentration is regulated at the levels of catabolism, inter- and intracellular transport, and biosynthesis.

Proline is synthesized from glutamate through reduction by pyrroline-5-carboxylate synthase (P5CS) and P5C reductase, and the rate-limiting step is catalysed by P5CS. *P5CS* gene expression is induced by environmental stress, and it is also under metabolic control, including negative feedback from proline itself. There are two P5CS enzymes in arabidopsis, which are highly expressed in the reproductive shoot apical meristem and floral meristems (Mattioli *et al*., 2009).

Proline is enriched in specific tissue types, regardless of its proportion relative to the other amino acids, with the highest concentrations in the flower, especially in the pollen grains and seeds (Schwacke *et al*., 1999; Verbruggen and Hermans, 2008). Proline-dependent osmoregulation is especially important for drought and salinity responses, or environmental stress conditions in general, including acidic conditions, low temperature and heavy metals (Delauney and Verma, 1993).

There are clear spatio-temporal specificities in osmoregulation. It is more active in young growing tissues than in more mature tissues. Under water stress, older leaves wilt first, while younger leaves stay turgid and even continue to grow (Molz and Boyer, 1978). Sugars and amino acids are major constituents of osmoregulation in most expanded leaves and growing hypocotyls. In growing organs, osmoregulation is largely dependent on import of solutes, in particular the products of photosynthesis. Changes in the concentrations of K^+^ and its counterions (e.g. malate and chloride) also contribute greatly (Morgan, 1984; Cazalé *et al*., 1998).

Each cell type has a unique composition of osmotica. Single-cell sampling of cellular sap, combined with pressure probe measurement, revealed four phases of osmolyte accumulation, depending on the main osmotica type, in the developing root of *Daucus carota* (Korolev *et al*., 2000). They were: an amino acid phase (in germinating seedlings); an ion phase (inorganic and organic ions: K^+^, nitrate and malate); a hexose phase (glucose and fructose); and a sucrose phase. The amino acid phase was specific to the germinating seedlings, whereas the latter three phases were observed in more mature, sweetening carrot root. Different cell types had different osmolyte compositions, and thus the different types of osmolyte are interchangeable with regards to turgor pressure. For example, sugar content was the highest in the cells near the vascular cambium, and the concentrations of K^+^ and sugar were usually reciprocal. Interestingly, the cambial cells contain exceptionally low K^+^ and sugar concentrations.

Furthermore, each cell type can utilize multiple mechanisms of osmoregulation. During the opening of stomatal guard cells, at least three alternative osmoregulation pathways are employed depending on the time of a day and the type of light (Talbott and Zeiger, 1998). The guard cells open when their turgor increases due to the surge in their osmotic potential and resulting water influx. For the osmoregulation in these cells, carbohydrates, as well as K^+^ and its counterions, are the main osmotica to be modulated. In red light conditions, photosynthetic sucrose production supplies the osmotica. In blue light conditions, there are two possibilities. In the earlier half of the opening period, starch is broken down into malic acid, while a high H^+^ concentration in the apoplast drives Cl^–^ and K^+^ influx into the cell. In the later half of the opening period, starch turns into sucrose to maintain the high osmotic potential and keep the guard cells open.

## QUANTIFYING TURGOR PRESSURE AND PLANT HYDRAULICS

In order to assess the role of turgor pressure and its regulation, it is fundamental to obtain quantitative data on water potential, hydrostatic (turgor) pressure, osmotic pressure and hydraulics, which we review in the following. All definitions of technical terms are given in Appendix 1, together with the laws of physics that are needed to estimate them. Briefly, water flows from high to low water potential. Water potential is generally the sum of two contributions: the osmotic potential *Ψ_Π_* and the pressure potential *Ψ*_p_, *Ψ*_w_ = *Ψ_Π_* + *Ψ*_p_. The pressure potential value is simply given by the hydrostatic pressure *P* (relative to atmosphere), *Ψ*_p_ = *P*. The osmotic potential value is given by the negative of osmotic pressure *Π*, *Ψ_Π_* = –*Π* = –*MiRT*, where *M* is the molarity of the solution contained in the compartment in mol L^–1^, *i* the Van't Hoff factor which represents the number of distinct particles produced when the substance is dissolved (e.g. *i* = 2 for NaCl, 1 for mannitol), *R* the gas constant (8·314 L kPa^–1^ K^–1^ mol^–1^) and *T* the absolute temperature (in Kelvin). Therefore, if a cell is at equilibrium with a bath of pure water at atmospheric pressure (*Ψ*_w_ = 0), then its turgor pressure is equal to its osmotic pressure, *P* = *MiRT*. Many of the methods below are based upon the generalization of this argument.

### Pressure measurements

We first survey the techniques that give access to the quantification of turgor pressure, from the organ level down to the cell level.

#### Psychrometer

**Fig. 2.**
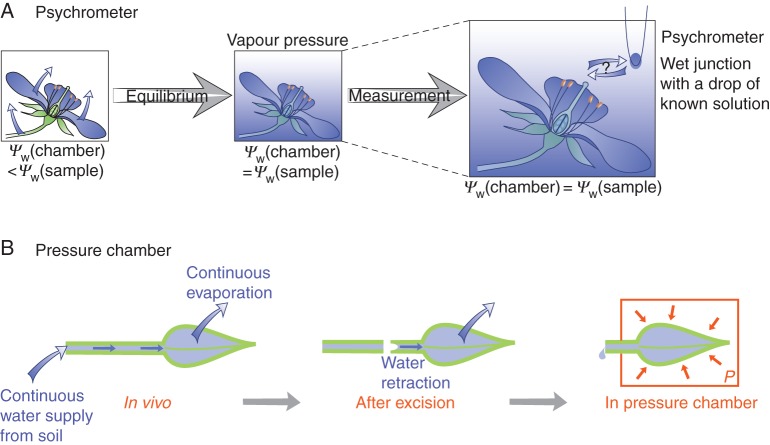
Measurements of water potential and hydraulic conductivity at the organ scale. (A) Psychrometer. (B) Pressure chamber.

A psychrometer can be used to measure average turgor in a tissue or an organ. Its modern form is due to Boyer and collaborators (Boyer and Knipling, 1965; Boyer, 1966). A tissue or an organ is put in a small closed chamber (Fig. 2A). Water evaporates from the sample until saturation vapour pressure is reached (note that the amount of water that has evaporated is small with respect to the amount of water in the sample, and so the change in water content of the sample is negligible). The system is then at equilibrium: water potentials are equal in the sample and in the gas phase; evaporation and condensation of water compensate each other, ensuring a steady state.

A thermocouple (an electric circuit such that a temperature difference yields a voltage) is then used to measure the water potential. A small drop of a solution of known osmolarity is put on the wet junction of the thermocouple, and the thermocouple is inserted into the chamber. If the drop has a lower water potential than the chamber, then some water condenses from the atmosphere onto the drop, inducing a local temperature increase, which can be detected in comparison with the dry junction. If, conversely, the drop has a higher water potential, then some water evaporates from the drop. A drop of isopiestic solution, i.e. having the same vapour pressure as the chamber, does not induce a temperature change. The water potential of the sample is then equal to the water potential of the isopiestic drop which can be easily calculated from its osmolar content (see ‘Water potential’ in Appendix 1). To access turgor pressure in the organ, the osmotic pressure of the tissue cells is required, which can be obtained by extracting cell sap (e.g. by freezing, thawing and putting the sample in a syringe) and measuring its water potential with the same approach (or with other approaches, see below). An estimation of the average turgor of the sample, *P*(sample), is then the sum of the osmotic pressure *Π*(sample) and the water potential *Ψ*_w_(sample). With this technique, turgor pressure was found to be 0·46 MPa in the soybean stem (Nonami *et al*., 1987) and 0·18 MPa in arabidopsis intermediate leaves (Hayot *et al*., 2012).

#### Pressure chamber

A pressure chamber (also known as the pressurized chamber or the pressure bomb) is used to measure turgor in the excised part of a plant (Scholander *et al*., 1964; Boyer, 1995). For instance, the leaf is cut and placed in a closed chamber with the petiole emerging from the chamber through a sealed joint so that the cut remains outside (Fig. 2B). By increasing the pressure *P*(chamber) inside the chamber until the sap start to exude, it is possible to determine the water potential of the tissue cells.

The protoplastic (p) and apoplastic (a) domains of the sample are in equilibrium. The protoplastic domain, which includes all the cell content, has an additional hydrostatic pressure *P*(chamber), whereas the apoplastic domain, defined by the rest of the plant, cell wall and xylem, is at the reference atmospheric pressure because the cut is in contact with the outside. Equilibrium yields *Ψ_Π_*(p) + *Ψ*_p_(p) + *P*(chamber) = *Ψ_Π_*(a).

*Ψ_Π_*(a) can be determined by collecting some sap and measuring its osmolarity. *P*(chamber) is known. The water potential of the cells *in vivo* is *Ψ_Π_*(p) + *Ψ*_p_(p) and can then be calculated. If the osmolarity of the cells is measured (see below), then their turgor pressure can be deduced. Scholander *et al*. (1964) found values in the range 0·45–0·6 MPa for twigs from various species.

**Fig. 3.**
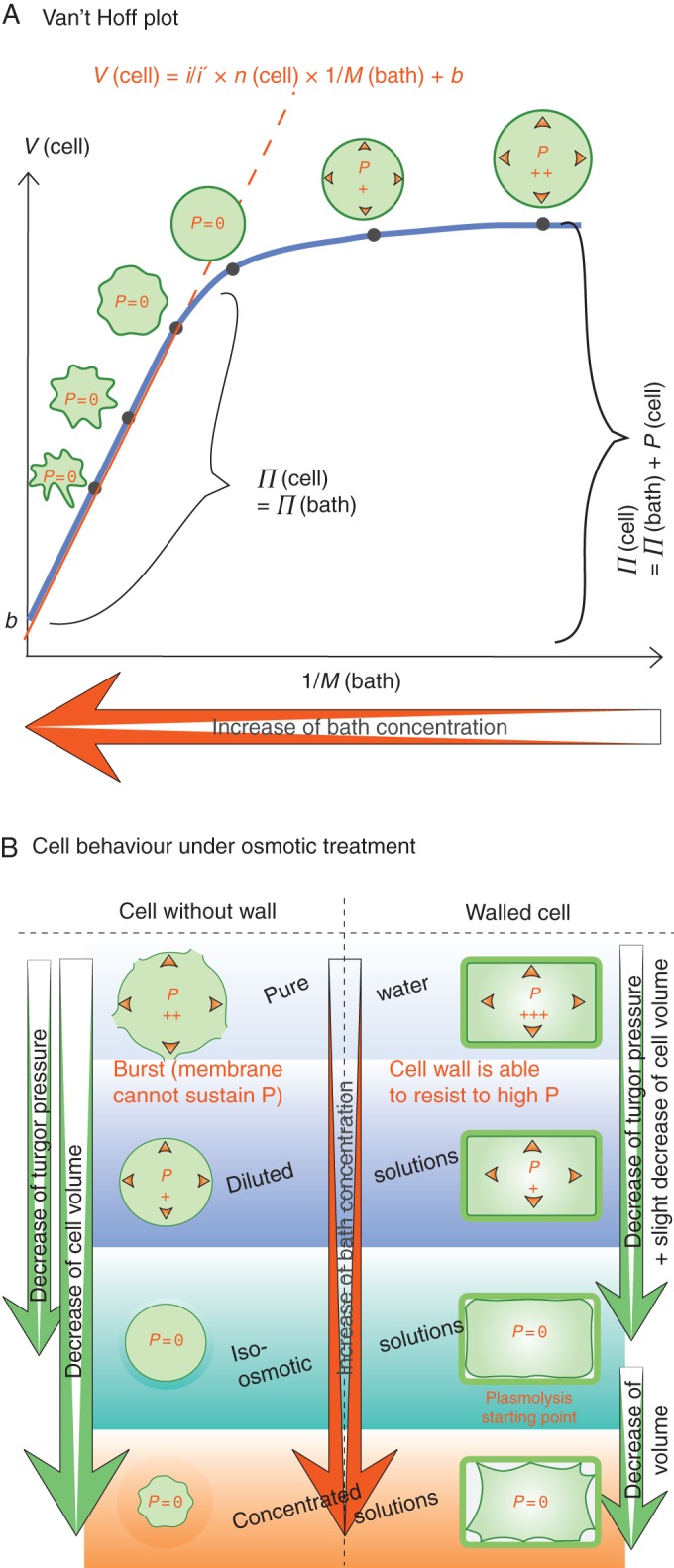
Measurement of turgor with osmotic treatments. (A) Van't Hoff (Höfler) plots that enable the determination of the cell solute concentration. (B) Schematics of cell deflation in hyperosmotic solutions.

We next consider the methods developed to obtain turgor at the cell level. The first two are based upon cell volume changes under osmotic treatments (Fig. 3B).

#### Boyle–van't Hoff plots

These plots were introduced by Höfler in 1920 (and are also known as Höfler diagrams) and their use was improved by Dainty (1972), Tyree and Hammel (1972) and Zimmermann *et al.* (1976); see Zimmermann (1978) for a review. The method consists of bathing an isolated cell, or a tissue, successively in a set of solutions of graded molarity, *M*(bath), and of measuring the volume of the protoplast (or a of a given protoplast in a tissue), *V*(cell), for each bath, as shown in Fig. 3A. The linear part of the plot, when there is no turgor pressure inside the cell [*P*(cell) = 0], allows the determination of the total quantity of solutes in the cell, *n*(cell). Indeed, in this region, equality of water potentials implies that *M*(cell)*iRT* = *M*(bath)*i'RT* (the same notations as above, for cell and bath, *i* and *i'* representing the respective Van't Hoff factors), and hence

$$V(\text{cell})=i/i^{\prime }\times n(\text{cell})/M(\text{bath})+b.$$

In this equation, *b* represents the hypothetical volume inside the cell that is inaccessible to water, and thus does not participate in the water potential. The slope gives access to *n*(cell) which is assumed to be constant (but see hereafter). When *n*(cell) is known, and since *V*(cell) can be read on the plot for each bath condition, *P*(cell) can be calculated. The main weakness of this technique is the assumption that *n*(cell) is constant (semi-permeable membrane and no active osmoregulation). Therefore, it would be expected that the osmotic pressure, and hence turgor, are overestimated. Also, a reliable optical measurement of volume is required for the method. The values found for turgor pressure are 0·5–0·8 MPa for the alga *Chlorella emersonii* (Munns *et al.*, 1983) and 0·8–0·9 MPa in *Allium cepa* (Gerdenitsch, 1984)*.*

#### Incipient plasmolysis

Incipient plasmolysis is closely related to the previous approach, but is applicable only to walled cells because it relies on the plasmolysis point (for comparison of behaviours of protoplast-like and walled cells when increasing their bath osmolarity, see Fig. 3B). In addition to the same questionable assumption that *n*(cell) is constant, it is considered that the cell wall is so stiff that the cell volume change is negligible between the turgid state and the onset of plasmolysis. In order to deduce their turgor pressure, cells are put in baths of increasing osmolarity. The iso-osmotic concentration is determined by the onset of plasmolysis. Then the cell osmotic pressure can be calculated as *Π*(cell) = *Π*(bath) = *M*(bath)*iRT*, hence turgor in any bath as above. This technique is easier to implement because volume measurement is not required, but the assumptions that the cell wall is infinitely stiff and that no osmoregulation occurs also lead to an overestimation of turgor. Moreover, any adhesion between the plasma membrane and the cell wall would delay the observation of plasmolysis. Indeed, the turgor pressure of 0·79 MPa found for the pollen tubes of *Lilium longiflorum* using incipient plasmolysis (Benkert *et al*., 1997) is significantly higher than that found with the pressure probe (see below).

#### Pressure probe

**Fig. 4.**
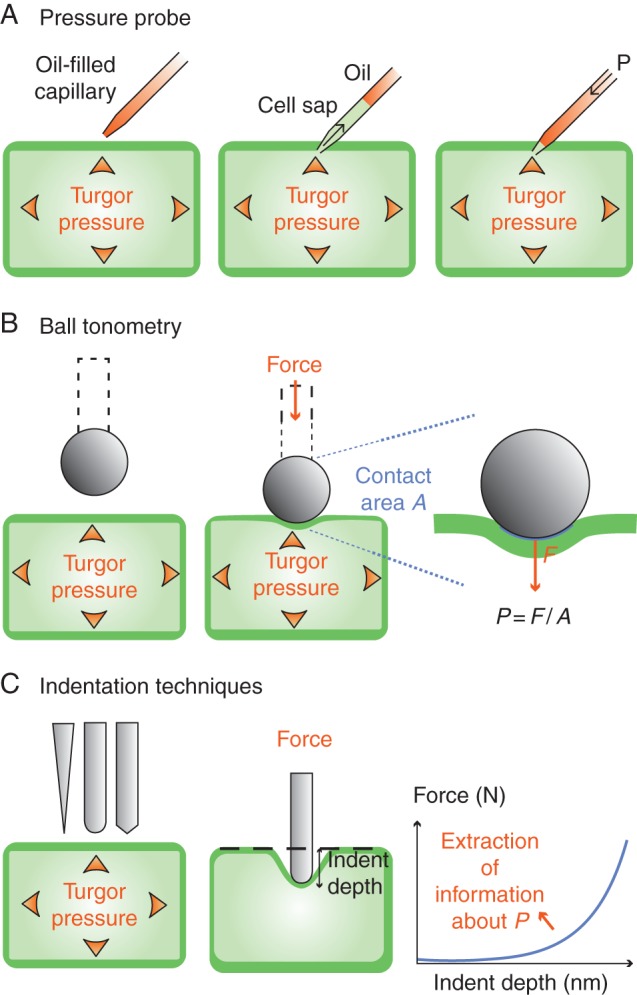
Measurement of turgor with mechanical methods. (A) Pressure probe, wherein the pressure is measured directly using a capillary. (B) Ball tonometry, wherein the surface of contact between the sphere and the cell is determined optically. (C) Indentation methods, wherein a force–depth curve is interpreted to yield the pressure.

A pressure probe enables a direct measurement of turgor in a specific cell, isolated or within a tissue. An earlier set-up consisted of a capillary inserted in internodal cells of *Nitella flexilis* that were emptied of their protoplasts (Kamiya *et al.*, 1963); the cell walls were pressurized using a syringe connected to this capillary, the pressure being measured with a Bourdon-type manometer. In the first version of the pressure probe (Green, 1968), a small capillary filled with water and containing a bubble of air was inserted into a living cell. By monitoring the volume change of the bubble, it was possible to deduce the pressure acting on it, i.e. turgor pressure. The method was then improved (see, for example, Hüsken *et al*., 1978; Tomos and Leigh, 1999) by filling the capillary with oil connected to an oil chamber with a pressure sensor. When the capillary is inserted into the cell, cell contents enter the capillary because of the higher hydrostatic pressure in the cell, and a visible cell sap–oil meniscus forms (Fig. 4A). A piston is moved in the chamber to bring back the meniscus to the position of the cell wall (the initial limit of the cell contents); the pressure measured then is exactly turgor pressure.

This method has been successfully applied to different cell types. Turgor was found to be equal to about 0·4 MPa in stems of *Glycine max* (Nonami *et al*., 1987), 0·4 MPa in trichome cells of *N. clevelandii* (Oparka and Prior, 1992), 0·4–0·6 MPa in *Z. mays* and *Triticum aestivum* roots (Rygol *et al*., 1993), 0·3 MPa in *S. lycopersicum* roots (Griffiths *et al*., 1996), 0·3–0·6 MPa in the arabidopsis root epidermis (Shabala and Lew, 2002; Javot *et al*., 2003), 0·2 MPa for lily pollen tubes (Benkert, 1997) – much lower than with incipient plasmolysis – 0·4 MPa in arabidopsis suspension cells (Gerbeau *et al*., 2002) and 0·3 MPa for tomato single cells (Wang, 2006). This technique also revealed radial gradients in the root, with turgor increasing from the epidermis to the vascular strand (Meshcheryakov *et al*., 1992; Zimmerman *et al*., 1992; Rygol *et al*., 1993), especially with enhanced transpiration. The pressure probe has two main drawbacks: it is intrusive and the size of the probe (about 4 μm tip diameter) does not allow its use on very small cells (such as meristematic or guard cells in many plant species).

#### Indentation methods

The principle of indentation methods stems from the observation that turgid plants appear stiffer than when flaccid. This should also apply to the apparent stiffness when pushing on (indenting) a cell with a probe (Fig. 4B, C). In ball tonometry (Lintilhac *et al*., 2000), a small glass bead (0·05–0·5 mm in diameter) is loaded with a controlled force onto an onion epidermal cell and the resulting contact area between the ball and the cell is optically measured. The pressure is then deduced using the relationship between pressure, force and area (see Fig. 4B), yielding a value of about 0·6 MPa. The principle is similar to the micromanipulation technique used in Wang *et al*. (2006), where a single cell is squeezed between a small cylindrical flat-ended probe and a flat surface, yielding a value of 0·3 MPa in tomato single cells. These approaches are efficient but the interpretation of the results discarded the contribution of the cell wall to cell stiffness.

The use of more refined instruments such as the atomic force microscope (AFM) or microindenters (such as the cellular force microscope; Routier-Kwierzkowska *et al*., 2012) provides the ability to control a very small tip (radius typically <1 μm) and push it into a particular cell (see Fig. 4C). As the indentation depth increases, the required force increases. Analysing the force–indentation curve with appropriate mechanical models makes it possible to take into account the contribution of the cell wall, but the deduction of the turgor pressure value is still an active research field (Deng *et al*., 2011; Forouzesh *et al*., 2013; Routier-Kwierzkowska *et al*., 2012; Vella *et al*., 2012*a*, *b*). With such a method, Vogler *et al*. (2012) obtained values of 0·3 MPa for lily pollen tubes, much closer to pressure probe measurements than incipient plasmolysis (Benkert *et al*., 1997).

### Osmolarity measurements

Among the techniques described above for turgor pressure, some also enable determination of the osmolarity of the sample: e.g. Höfler diagrams and the incipient plasmolysis technique, since knowing the osmolarity is a prerequisite for the determination of turgor; or the psychrometer as the isopiestic drop in equilibrium with the solution inside the chamber has the same osmolarity as the solution.

In freezing-point osmometry, the cell content needs to be extracted. The osmometer will cool it until it freezes. The presence of solutes in a solvent reduces its freezing point. (It is common practice during winter to put salt on roads because salt lowers the freezing point.) This phenomenon depends mostly on the concentration of solutes and not on their nature, at least for small solutes [for large solutes such as polyethylene glycol (PEG), osmotic potential depends non-linearly on concentration]. By measuring the freezing point of the solution, the osmometer gives access to its osmolarity. Osmolality (per kg, unlike osmolarity which is per L) of barley leaf epidermal cells was measured from 430 to 550 mosmol kg^–1^ with this technique (Fricke *et al*., 1994).

### Water and solute movement

The first methods at the organ level are based on imposing or measuring a water flux and its proportionality with differences in pressure or in water potential.

#### Pressure chamber

It is possible to deduce the hydraulic conductivity *L*_p_ (see Appendix 1) with a pressure chamber (Boyer, 1995), using the relationship *L*_p_ = *J*_v_/(*A* × *P*) where *J*_v_ is the rate of sap flow that is exuded from the excised tip because of the pressure, *P*, applied inside the chamber and *A* is the overall surface area of the sample. Values of *L*_p_ found with this technique are about 2 × 10^–8^ m s^–1^ MPa^–1^ for the root of *Lotus japonicus* (Henzler *et al*., 1999), 2·7 × 10^–6^ m s^–1^ MPa^–1^ for arabidopsis root and 1·5 × 10^–7^ m s^–1^ MPa^–1^ for arabidopsis rosette (Postaire *et al*., 2010).

#### Vacuum pump

In this method (Kolb *et al*., 1996), a piece of plant is enclosed in a chamber with negative pressure *P*, the stem or the petiole being outside the chamber and immersed in water. The negative pressure pulls water from the bath through the plant with a flow rate (*F*, units mmol s^–1^ m^–2^ or kg s^–1^ m^–2^) that depends on the hydraulic conductance of the sample, *K*. The value of *K* is given by the slope of the flow rate vs. the vacuum pressure, *P*. This technique gave values in the range 0·4–0·8 mmol s^–1^ MPa^–1^ for the root of *Artemisia tridentata* (Kolb *et al*., 1996).

#### High pressure flowmeter

In this method introduced by Tyree *et al*. (1995), pressurized water is forced into the stem of an excised plant towards the roots or leaves. The pressure (*P*, units MPa) at the inlet is tuneable, and the corresponding inflow (*F*, units mmol s^–1^ m^–2^ or kg s^–1^ m^–2^) can be measured. The slope of the *F* vs. *P* plot corresponds to the hydraulic conductance *K*. Typical values found for conductance range from 3 to 10 × 10^–4^ kg s^–1^ m^–2^ MPa^–1^ for leaves of different crop species (Tsuda and Tyree, 2000), and from 1 to 2 × 10^–4^ kg s^–1^ m^–2^ MPa^–1^ for mature leaves of different tropical species (Tyree *et al*., 2005). We note that the three previous methods tend to induce stomatal opening and are therefore more sensitive to inner conductance when leaves are probed, unlike the next method.

#### Evaporative flux

This method (Sack and Holbrook, 2006) is based on the proportionality between transpiration rate (*E*, units mmol s^–1^ m^–2^) and the difference in water potential between plant and soil, respectively *Ψ*_w_(leaf) and *Ψ*_w_(soil). The coefficient of proportionality is the conductance *K* = *E*/[*Ψ*_w_(soil) – *Ψ*_w_(leaf)]. Values of *K* found with this technique are in the range 1–10 × 10^–4^ kg s^–1^ m^–2^ MPa^–1^ for leaves of different crop species (Tsuda and Tyree, 2000), in agreement with the high pressure flowmeter (see paragraph above), and about 2 × 10^–4^ kg s^–1^ m^–2^ MPa^–1^ for the arabidopsis leaf (Martre *et al*., 2002). Values obtained with the three previous methods (vacuum pump, high pressure and evaporative flux) are in good agreement with each other because they are based on the same principles (Sack *et al*., 2002). The last method is less intrusive than the two previous ones, but measurements are more delicate.

#### Pressure probe

The pressure probe can be used to modulate the position of the meniscus and follow the dynamics of the pressure until a steady state is reached, with a half-time *τ*. The hydraulic conductivity of the cell membrane, *L*_p_, is determined by *L*_p_ = ln2 × *V*(cell)/[*A*(cell) × *τ* × (*ϵ* + *Π*)] where *V*(cell) is the initial volume of the cell, *A*(cell) its membrane area, *ϵ* = *V* d*P*/d*V* (derivative of pressure with respect to volume) the volumetric elastic modulus, and *Π* the internal osmotic pressure. *ϵ* expresses how the volume of the cell changes with pressure due to cell wall elasticity, and this value is therefore accessible by applying a pressure difference and estimating the volume change by optical means (see, for example, Zimmerman *et al*., 1976).

The pressure probe can also be used to perform a ‘pressure clamp’ experiment (Wendler and Zimmermann, 1982): the pressure inside a cell is artificially maintained at a constant value higher than that at rest. The expression of *L*_p_ becomes *L*_p_ = – *S*_v_/[*A*(cell) × Δ*P*] where *S*_v_ is the initial slope of the volume relaxation [=(dΔ*V*/d*t*)|*_t_*_=__0_], *A* is the membrane area, and Δ*P* is the difference between the initial turgor pressure and the imposed pressure.

This is an improvement on the previous method to deduce *L*_p_ because there is no need to know *Π* and *V*(cell), which reduces errors. The volume change Δ*V* is imposed with the pressure probe, and therefore more precisely known than if measured optically. Typical values of *L*_p_ found with the pressure probe are about 1·7 × 10^–4^ cm s^–1^ MPa^–1^ for *Chara corallina* internodes cells (Zimmermann and Husken, 1979; Wendler and Zimmermann, 1982), 6 × 10^–7^ m s^–1^ MPa^–1^ for corn and barley young roots (Joshi *et al*., 2009), 9 × 10^–7^ m s^–1^ MPa^–1^ for maize young roots (Knipfer *et al*., 2007), 1·2 × 10^–8^ m s^–1^ MPa^–1^ for tomato roots (Griffiths *et al*., 1996), 5 × 10^–7^ m s^–1^ MPa^–1^ for root cortical cells of lotus (Henzler *et al*., 1999), 4·6 × 10^–7^ m s^–1^ MPa^–1^ for arabidospsis suspension cells (Gerbeau *et al*., 2002), and 0·5–8 × 10^–6^ m s^–1^ MPa^–1^ for parenchyma cells of Venus flytrap leaf (Colombani and Forterre, 2011). Note that in techniques where the volume of displaced water is important, such as in the pressure clamp or the high pressure flowmeter, solutes can accumulate in front of the osmotic barrier so that the compartment is no longer at osmotic equilibrium. This can lead to significant errors in the calculated value of *L*_p_ (Knipfer *et al*., 2007).

Finally, the pressure probe can also be used to monitor solute exchange indirectly (Steudle *et al*., 1987). When the external bath of a maize root was changed to a bath containing a high concentration of solute, the pressure measured was observed to follow a fast relaxation, probably corresponding to water movement from the root to the bath, and a slow secondary relaxation, that could be ascribed to ion exchanges between the root and the bath. As osmoregulation cannot be excluded, the time scale of the secondary relaxation could correspond to the half-time of ion exchange or of osmoregulation (or of a combination of both).

#### Specific ion electrode technique/scanning ion-selective electrode technique/microelectrode ion flux estimation/ion-specific vibrating probe

This technique can be used to quantify the concentration and flux of specific ions. It involves the measurement of the voltage difference between a reference electrode and an electrode in contact with the solution of interest and filled with an ionophore, i.e. a lipid-soluble compound that is only permeable to a specific ion. The voltage difference is related to the concentration of this ion by the Nernst equation. By displacing the measurement electrode, it is possible to deduce the gradient of this ion close to the cell, and hence the diffusive flux across the surface using Fick's law and a tabulated value of the diffusion coefficient (Kochian *et al*., 1992) or a calibration in known ion gradients (Kühtreiber and Jaffe, 1990). In practice, this electrode is oscillated sinusoidally, allowing the measurement to be quicker than the system drift (Kühtreiber and Jaffe, 1990; Kochian *et al*., 1992). Using this technique, it was found that fluxes had values of about 30 pmol cm^–2^ s^–1^ for Ca^2+^ influx at the cell apex of cotton fibre (Tang *et al*., 2014), whereas they ranged from 100 to 500 pmol cm^–2^ s^–1^ for Na^+^ efflux in the root of cucumber and pumpkin (Lei, *et al*., 2014). However, a main drawback of this technique is the poor specificity of some ionophores, as shown for chloride (Messerli *et al*., 2004).

#### Fluorescent tracing

Indications on solute movement of larger molecules and therefore plasmodesmatal connectivity can be obtained by monitoring the movement of a fluorescent dye in the tissue. Since membrane channels do not generally allow the passage of such dyes, fluxes can be ascribed to plasmodesmata. Dye passage can therefore be ascribed either to simple diffusion or to mean water flow across plasmodesmata. The dye can be microinjected in a specific domain (Han *et al*., 2014), or loaded by iontophoresis (application of a current of a few nanoAmps through the membrane to facilitate the entry of the dye) as in Rinne and van der Schoot (1998). Plants can be bombarded with microparticles carrying plasmids with *GFP* (green fluorescent protein) constructs (Liarzi and Epel, 2005). In other experiments, all the tissue is dyed but a restricted zone is photobleached, allowing the measurement of the recovery time (Rutschow *et al*., 2011). These methods allow the deduction of the effective diffusion constants between cells inside the same symplastic domain and the plasmodesma permeability; however it is not possible to distinguish between water flow and solute diffusion. Typical values for arabidopsis root are *D* approx. 50 μm^–2^ s^–1^ and permeability approx. 10 μm s^–1^ (Rutschow *et al*., 2011).

## CASE STUDIES

**Fig. 5.**
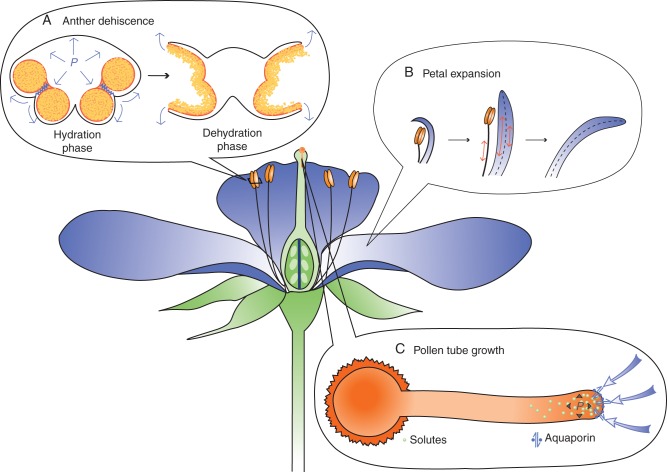
Case studies in the flower. (A) Anther dehiscence. (B) Petal expansion. (C) Pollen tube growth.

Based on the information we have described above, we now illustrate how changes in turgor pressure and different mechanisms of turgor modulation drive developmental processes, drawing three major events in flower development as examples (Fig. 5).

### Anther dehiscence

When plants are ready for reproduction, the anthers open to expose and release mature pollen. This process is called anther dehiscence, and in many species it is the temporal determinant of pollination and subsequent fertilization. Anther dehiscence is thought to result from a refined sequence of water allocation within the stamen (Fig. 5A); the endothecium (the sub-epidermal tissue) and other specific cell types are actively hydrated first and then dehydrated during the final stages of stamen differentiation (Scott *et al*., 2004; Nelson *et al*., 2012).

During the hydration phase, water fluxes into the endothecium and pollen inflate these structures. The enhanced hydration of the epidermal and sub-epidermal endothecium cells increases up the turgor pressure in these cells and the turgor-driven force borne by the epidermis, dramatically increasing the lateral tension in the epidermis. The heightened tension ruptures the weakest point – the stomium cells – and breaks open the entire anther locules (Keijzer *et al*., 1987).

Osmoregulation through K^+^ accumulation seems to be the key in turgor control en route to anther dehiscence. The Na^+^/H^+^ antiporters NHX1 and NHX2 are necessary for maintenance of proper vacuolar pH and K^+^ concentration. In *nhx1; nhx2* double mutants of arabidopsis, some flowers (approx. 7 %) have problems with stamen filament elongation and anther dehiscence (Bassil *et al*., 2011). In barley, K^+^ is highly concentrated especially in the stomium; this cell-specific K^+^ accumulation may lead to targeted turgor enhancement and then breakage (Rehman and Yun, 2006). In *Oryza sativa*, on the other hand, it has been reported that swelling of pollen due to specific K^+^ accumulation there is responsible for stomium breakage (Matsui *et al*., 2000).

Once the stomium breaks, the anthers start to dehydrate. The endothecium has string-like secondary thickening of the cell wall that is mainly composed of lignin. When those cells dry, the endothecium undergoes enhanced shrinkage like a spring and creates an outward bending force, leading to full opening of the anthers and effective pollen release (Keijzer *et al*., 1987). MYB26 transcription factor is necessary and sufficient for the secondary thickening of the endothecium cell wall in arabidopsis. The mutant of the gene (*male sterile 35*) can open the anther only partially, even though the stomium is broken (Yang *et al*., 2007). PIP2 aquaporin proteins, which are specifically expressed in the anthers and styles, facilitate the withdrawal of water in tobacco (Bots *et al*., 2005*a*, *b*). The anthers of RNAi (RNA interference) plants with reduced expression of *PIP2* genes dehisced more slowly compared with the wild type; nuclear magnetic resonance (NMR)-based tracking of water movement indicated slower water removal from the anthers.

Anther dehydration is also driven by accumulation of osmolytes in the surrounding connective tissues and stamen filaments. The sucrose transporter AtSUC1 is localized in the connective tissues, which could drive water out of the anthers (Stadler *et al*., 1999). The *DAD1* (*DEFECTIVE IN ANTHER DEHISCENCE1*) gene of arabidopsis, which encodes the enzyme that catalyses the first step of jasmonic acid (JA) biosynthesis, is expressed in the filaments immediately prior to flower opening (Ishiguro *et al*., 2001). The *dad1* mutant has defects in anther dehydration and is delayed in anther dehiscence. The mutant is reduced in filament elongation and petal expansion, and thus a model was proposed for hydraulic synchronization of anther dehiscence and flower opening. Translocation of water from the anther to the filament and petal would ensure that a suite of pollination-related events occurred simultaneously. The model is in accordance with the finding in *Allium cepa* that filament elongation occurs concomitantly with anther dehiscence (Keijzer *et al*., 1987).

### Flower opening

In addition to anther dehiscence, opening of the flower generally marks the onset of pollination. Flower opening is thought to facilitate pollen maturation and release, and it also makes the stigma on the carpels accessible to pollination (van Doorn and van Meeteren, 2003). There are several types of flower opening (e.g. single vs. repeated or nocturnal vs. diurnal) depending on the species; most species, however, undergo single and permanent opening.

Water movement is important for opening of the flower, since the process is impaired in rose cut flowers if there is a blockage in the basal stem (van Doorn and van Meeteren, 2003). In some other species (e.g. cotton and chrysanthemum), the continuum of water in the flower is separated from that in the stem, and the flower can open even when leaves are wilting due to desiccation (Trolinder *et al*., 1993; van Doorn and van Meeteren, 2003). Such separation may be highlighting the importance of water relations in flower opening and development in general.

Floral buds open due to petal expansion and, to a lesser extent, stamen elongation (Fig. 5B). Flower opening is typically rapid and is completed within 5–30 min. In most species it occurs due to uncovering of the petals (i.e. removal of physical constraints, for example through the abscission of bracts and sepals) or petal movements. Petal movements can be controlled by cell expansion or shrinkage via osmoregulation. For example, the petals of *Gentiana kochiana* and possibly *Kalanchoe blossfeldiana* move through reversible, turgor-driven expansion and contraction of cells on the adaxial surface (van Doorn and van Meeteren, 2003). A similar mechanism of cell expansion/shrinkage leading to reversible opening of a structure also underlies the opening/closure of the ice plant seed capsules (Harrington *et al*., 2011).

In most species studied to date, petal movements occur due to differential growth rate of the epidermis on the two sides (i.e. abaxial or adaxial sides). A flower opens when the adaxial side of petals grows more than the abaxial side (Fig. 5B). This differential growth is mostly mediated by differential osmoregulation; carbohydrates accumulate, from either mobilization of storage carbohydrates or sucrose import (van Doorn and van Meeteren, 2003). Young flowers typically contain high levels of starch. Solute levels increase prior to flower opening through the uptake of sugars from the apoplast and the conversion of polysaccharides (starch, fructan or both) to monosaccharides (fructose and glucose). Supporting this notion, inhibition of starch degradation prevented petal growth in lily (Bieleski *et al*., 2000).

The petals of Morning Glory (*Ipomoea tricolor*) open early in one morning and senesce in the afternoon of the same day. The senescence starts by rolling of the corolla, curling the rib starting from the distal tip margins. This process is induced by ethylene, which enhances ion (Rb^+^) and sucrose efflux from the rib; the rolling could be due to asymmetric turgor changes, since the petals could be unrolled when turgor pressure was eliminated in a strong hyperosmotic treatment that plasmolysed cells (Hanson and Kende, 1975).

Aquaporins play central roles in petal cell expansion, and they mediate ethylene-dependent inhibition of flower opening. In roses, ethylene inhibits or promotes petal growth depending on the cultivars (Reid *et al*., 1989), and in the cultivar ‘Samantha’ it negatively regulates petal expansion. Ethylene treatments resulted in irregular, smaller petals due to less cell expansion and water content, and aquaporin genes *PIP1;1* and *PIP2;1* were found to be downregulated (Ma *et al*., 2008; Chen *et al*., 2013). Ethylene seems to regulate aquaporin expression and ultimately petal cell expansion, via miRNA164-mediated post-transcriptional regulation and the NAC-domain transcription factor RhNAC100 (Pei *et al*., 2013).

In the case of tulip, in which the flower opens diurnally according to the daily temperature fluctuations, petal expansion is regulated at the level of aquaporin protein activity. The petals open when the temperature rises in the morning, and they close in the evening when the temperature drops. It has been shown that warm temperature activates water transport into the petal cells via the phosphorylation of PIP2;2 aquaporin in a Ca^2+^-dependent manner (Azad *et al*., 2004, 2008).

### Pollen tube growth

When pollen lands on the stigma at the tip of carpels, it bulges out at one position and begins to elongate as a tube. The pollen tube is the tubular extrusion of the vegetative cell of the microgametophyte. It carries two sperm cells inside and elongates inside the style of the carpels to bring the sperm to the megagametophyte for fertilization. The pollen tube is an autonomous single-cell system that undergoes localized growth at the very tip, a type of cell growth called ‘tip growth’ (Fig. 5C), which is also found in fungal hyphae and root hairs. The pollen tube is one of the best-studied systems in plant cell biology, especially with regards to the investigation of cell growth regulation.

Growth is thought to be driven by turgor pressure, and the pollen tube is one of the fastest growing cells (Sanati Nezhad and Geitmann, 2013). Thus a question arises: is turgor pressure particularly high in the pollen tube? AtSUC1 is highly expressed in pollen and important for pollen tube germination and growth (Stadler *et al*., 1999; Sivitz *et al*., 2008), suggesting the importance of sucrose import in pollen tube growth. Pollen has high contents of proline and hydroproline (0·14 % of the soluble molecules), which are enriched at the tip (Dashek and Harwood, 1974). As plasmolysis starts at the tip upon a strong hyperosmotic treatment (Hill *et al*., 2012), it might also be argued that water influx/efflux is restricted to the growing tip; it should be noted, however, that plasmolysis usually start at the corners of cells.

Benkert *et al*. (1997) conducted pressure probe measurement of lily pollen tubes, being able to keep a micropipette inserted for 20–30 min without affecting the growth significantly. The turgor was measured in the range 0·1–0·4 MPa, about 0·21 MPa on average. They also estimated the turgor pressure within the tube using incipient plasmolysis and found the mean to be 0·79 MPa. This discrepancy might be ascribed to the limitations of the incipient plasmolysis technique, e.g. cells could osmoregulate upon hyperosmotic treatments. Indeed the value 0·3 MPa obtained by Vogler *et al*. (2012) using microindentation is consistent with the pressure probe measurement. Benkert *et al*. (1997) found no clear correlation between growth rates and turgor pressure; faster growing tubes did not necessarily contain higher turgor.

This observation questions the classic assumption that turgor pressure is the driving force for growth. Such a driving force could be provided by callose deposition at the rear of the protoplast, pushing it forward (see, for example, Parre and Geitmann, 2004) and eventually forming callose plugs preventing backward motion, though deposition would need to be fast enough to account for the speed at which tubes elongate. The assumption that turgor pressure is the driving force can be further explored in the context of oscillatory growth of pollen tube. A pollen tube starts out growing steadily, but frequently switches to oscillatory growth over time, when it grows too quickly or experiences instabilities (Chebli and Geitmann, 2007). The tube cycles between fast and slow phases of growth, repeating the shifts between growth promotion and inhibition every few minutes; thus oscillatory growth allows fine dissection of the temporal sequence of factors acting on the growth regulation. The concentrations of osmotically active solutes (such as prolines) do fluctuate depending on the phases of the oscillatory growth (Daseck and Harwood, 1974). Ion influx (K^+^ and Ca^2+^) oscillates too, but its peak immediately precedes the slow growth phase, rather than the fast growth phase (Chebli and Geitmann, 2007). Furthermore, no consistent oscillation-dependent variation in turgor pressure was measured in lily pollen tube (Benkert *et al*., 1997).

Altogether, it is difficult to determine whether, in the pollen tube, turgor pressure drives growth in a dose-dependent fashion. Since plasmolysed cells that lack turgor pressure cannot grow, it can be said that a threshold turgor pressure is necessary for growth (Kroeger *et al*., 2011). On the other hand, excessive turgor pressure (around twice as much as normal) burst the tube at the tip (Benkert *et al*., 1997). Therefore, the role of turgor in growth may be that its calibration to a particular range is important for growth maintenance. Recently a model was proposed to explain such turgor calibration (Hill *et al*., 2012). An osmosensor detects the difference in osmotic pressure between the cytoplasm and apoplast. The osmosensor is localized to the plasma membrane at the growing tip of the tube to regulate the influx of water. Since it was found to be sensitive to the treatment with mercury (an inhibitor of aquaporins), an aquaporin was suggested to be the osmosensor (Shachar-Hill *et al*., 2013). Alternatively, in such fast-growing cells as the pollen tube, cell wall extensibility could be large, so that turgor would remain close to the yield pressure required for growth (Lockhart, 1965), which would explain why no oscillations of turgor have been observed. Changes in growth rates would be caused by any change in hydraulic conductivity or in osmotic potential, with no need to modulate turgor pressure. Nevertheless, turgor pressure would drive growth as in other scenarios.

## DISCUSSION

Turgor pressure tends to be taken for granted. It is perhaps too often believed to be passive and unresponsive. However, in reality, it is active and dynamic, constantly changing depending on internal and external cues. Despite the widely held dogma that turgor pressure is uniform in all cells of an organ (due to the numerous symplasmic connections, plasmodesmata), cells are equipped with machinery to create differential turgor pressure. In normal and stress-response processes of development and physiology, cells are constantly regulating their turgor pressure via molecular changes that modulate water influx/efflux into and out of the cell.

An emerging picture is that the regulation of turgor pressure and water movement in and out of the cell is critical for many developmental processes, in flowers and elsewhere. A series of recent reports on arabidopsis lateral root development collectively signify this point. At the stage of primordia bulging (stage IV), both paths of water movement (i.e. aquaporins and plasmodesmata) are restricted. Most *PIP* genes are downregulated, and altered levels of *PIP2;1* transcripts delayed lateral root emergence (Peret *et al*., 2012). Immature lateral roots are symplastically isolated in stages IV and onward; without this symplasmic separation, lateral root number and spacing were disturbed (Oparka *et al*., 1995; Benitez-Alfonso *et al*., 2013). Compression by mechanically stiff neighbouring cells is critical for lateral root emergence and development (Lucas *et al*., 2013; Vermeer *et al*. 2014), and inhibition of water movement into and out of the primordia would strengthen this compression effect. Whether such hydraulic isolation results in differential turgor or not remains to be elucidated. At least, it appears that turgor decreases in the root cortex following auxin treatment (Peret *et al*., 2012).

Despite many indications that it is possible and probably commonplace, differential turgor pressure remains rather elusive, since it is difficult to be measured directly. Technical advances in turgor measurement (precise, accessible to the deep tissue and non-destructive) are necessary for clear evidence of turgor variation. Fine-tip pressure probing or nano-indentation has the potential to deliver such a measurement strategy. In parallel to the mechanical measurement, an optical approach should also be employed; pressure-sensitive proteins, including fluorescent markers, can be engineered as biosensors of turgor pressure (Barstow *et al*., 2008, 2009; Watanabe *et al*., 2013).

Future technologies will facilitate investigation of turgor regulation and its functional significance. For example, the recently available lines of arabidopsis in which plasmodesmata can be closed in a cell type-specific manner (Vaten *et al*., 2011) are powerful genetic tools; they can reveal the importance of symplasmic movements of small molecules, including water. As we understand more about gating mechanisms of plasmodesmata, inducible plasmodesmata opening lines may become available, allowing us to dissect the importance of distinct symplasmic domains that pre-condition differential turgor pressure. Similarly, specific and controlled modulation of aquaporin function through drug treatments or genetic modification would greatly help the dissection of water movement among cells. Improved, cellular and sub-cellular resolution osmometer measurement and visualization of water content with magnetic resonance imaging (MRI) would also provide critical information underlying turgor differences and changes.

Once we can experimentally monitor and control the turgor pressure of specific cells, the importance of dynamic turgor regulation in plant development, growth and physiology will become more evident. Experimental data from physico-biological investigations often require interpretation with computer modelling, via close collaborations across biology and physical sciences. Theoretical and empirical efforts will together uncover how turgor drives morphogenesis by pressuring the cells, one cell at a time.

## APPENDIX 1

Brief definitions of turgor-related terms and the corresponding mathematical definitions are given below.

*Water potential* (of a specific compartment) (notation *Ψ*_w_, units MPa): this is the energy per unit volume of water in a specific compartment. The lower the water potential of a compartment is, the more ‘favourable’ is this compartment for water molecules. If possible, water flows towards compartments where the water potential is the lowest. This rule drives the osmosis phenomena. Water potential can be expressed as a sum of different factors involving gravity, interaction with the matrix, interaction with solutes and hydrostatic pressure (Boyer, 1995). In practice, this sum is often reduced to its two main components: the osmotic potential *Ψ_Π_* and the pressure potential *Ψ*_p_.

$$\Psi_{w}=\Psi_{\Pi }+\Psi_{p}$$

The water potential of a compartment containing only pure water at atmospheric pressure is zero (because both *Ψ_Π_* and *Ψ*_p_ are equal to zero). If two different compartments (e.g. a protoplast and its external bath) are at equilibrium — in other words there is no water movement — their water potentials are equal.

$$\Psi_{w}(\text{cell})=\Psi_{w}(\text{bath})$$

This relationship is very useful to deduce the properties of the cell, since the water potential of a bath solution can be measured with an osmometer or imposed by the solute content. The reference pressure potential of a solution under atmospheric pressure being zero, its water potential is equal to its osmotic potential. At equilibrium:

$$\Psi_{w}(\text{cell})=\Psi_{\Pi }(\text{bath})+\Psi_{p}(\text{bath})=\Psi_{\Pi }(\text{bath})$$

*Osmotic potential* (of a specific compartment) (notation: *Ψ_Π_* or more rarely *Ψ*s, units MPa): this energy per unit volume corresponds to the stabilization of water molecules by interacting with solutes. Therefore, the osmotic potential is always negative (stabilization), being equal to zero for pure water (taken as a reference). The osmotic potential value is given by the negative of osmotic pressure *Π*.

$$\Psi_{\Pi }=-\Pi =-MiRT$$

where *M* is the molarity of the solution contained in the compartment in mol L^–1^, *i* is the Van't Hoff factor (the product *M* × *i* is in other words the osmolarity of the solution), *R* is the gas constant (8·314 L kPa^–1^ K^–1^ mol^–1^) and *T* the absolute temperature. Equivalently, one can use the correspondence between *Π* = 0·1 MPa and *M'i* = 42 mosmol kg^–1^ (which holds at 20 °C).

*Osmotic pressure* (of a specific compartment) (notation *Π*, units MPa): contrary to common belief, it is not a physical pressure, but rather an expression of the osmolarity of the solution contained in the compartment, since *Π* = *MiRT* (see ‘Osmotic potential’). This common confusion comes from (in addition to the name ‘osmotic pressure’, which is confusing by itself) the statement that the osmotic pressure is the pressure that needs to be applied to a solution to prevent inward flow of water across a semi-permeable membrane. This is true but confusing. Consider a cell bathed in pure water. The water potential of the cell is *Ψ*_w_(cell) = –*Π* + *P*. The water potential of the pure water bath is zero. To prevent any water movement, the system should be at equilibrium, which means *Ψ*_w_(cell) = *Ψ*_w_(pure water) = 0. This implies *Π* = *P*. So if, numerically, the value of the osmotic pressure of the cell is the same as the value of its hydrostatic pressure, then there is no water movement. However, this is only true for a pure water bath, and this does not mean that osmotic pressure is an actual pressure, nor that high osmotic value implies high hydrostatic value. Put in a bath with high osmolarity, at equilibrium the hydrostatic pressure of the same cell will be low if not zero.

*Pressure potential* (of a specific compartment) (notation *Ψ*_p_, units MPa): this energy per unit volume corresponds to the stabilization or destabilization of water molecules due to the pressure inside the compartment. Atmospheric pressure can be taken as a reference. For pressures higher than atmospheric, ‘pressurized’ water molecules are destabilized (*Ψ*_p_ > 0). For pressures lower than atmospheric, water molecules are stabilized (*Ψ*_p_ < 0). This last case can only be achieved under specific conditions, for example in the xylem where the sap is in tension (negative pressure). Xylem therefore acts like a water pump system. The pressure potential value is simply given by the hydrostatic pressure *P* (relative to atmosphere).

$$\Psi_{p}=P$$

*Turgor pressure* = *hydrostatic pressure* (notation *P*, units bars or MPa): this is the force per unit area (Newton per square metre = Pa) water molecules apply on their environment.

*Vapour (saturation) pressure* (defined for a given temperature): this is the pressure in a gas when this gas and its liquid phase are at equilibrium. For each solution (with a specific water potential), there is a corresponding saturation vapour pressure.

*Permeability* (often expressed in μm s^–1^): a speed representing how fast a molecule (often a dye) can move through a barrier (e.g. a plasmodesma). It is sometimes used with the meaning of conductivity.

*Hydraulic conductivity* (notation *L*_p_, units m s^–1^ MPa^–1^ or μL s^–1^ m^–2^ MPa^–1^): the ratio between the flow rate of water, normalized by the area upon which this flow occurs, between two compartments and the difference in water potential between these two compartments. The compartments can be individual cells [the term cell hydraulic (or water) conductivity is then used, written *L*_pcell_] for which the area is often that of the plasma membrane, or tissues [e.g. for root, the term hydrostatic root hydraulic (or water) conductivity is used, written *L*_pr-h_]. In the case of cells, it will depend, for instance, on the concentration and activity of aquaporins.

*Hydraulic conductance* (notation *K*, units d mmol s^–1^ MPa^–1^ or kg s^–1^ MPa^–1^ or m^3^ s^–1^ MPa^–1^): the same as hydraulic conductivity (applied to water quantity, mass or volume), but with no normalization by area. This is a property of the organ or of the leafy shoot that is tested.

*Flux* (*J*, expressed in mol m^–2^ s^–1^ or in kg m^–2^ s^–1^): represents how many molecules can cross a unit area (of cell wall, per example) per unit time.

*Drop in water potential related to water flow*: the flux is proportional to the conductivity and to the water potential difference between the two compartments, *J* = *L*_p_ Δ*Ψ*_w_. In out of equilibrium situations, water flows (driven for instance by evaporation), generating drops in water potential, and so in turgor, that depend on conductivity and on flux. Therefore, the regulation of conductivity can lead to modulations in turgor pressure.

*Flow rate* (*J*_v_, units m^3^ s^–1^ or μL s^–1^): represents the volume of liquid (e.g. sap) exuded from a cut per unit time.

*Half-time of water exchange* (*t_1_*_/2_, units s): after a rapid increase or decrease of the pressure inside a cell (e.g. forced with a pressure probe), water exchanges with the surrounding cells allow the pressure to stabilize again at its steady-state value. The pressure follows an exponential behaviour as a function of time, and *t*_1/2_ is the time needed to recover half of the pressure difference.

## References

[MCU187C1] Aharon R, Shahak Y, Wininger S, Bendov R, Kapulnik Y, Galili G (2003). Overexpression of a plasma membrane aquaporin in transgenic tobacco improves plant vigor under favorable growth conditions but not under drought or salt stress. The Plant cell.

[MCU187C2] Apse M, Aharon G, Snedden W, Blumwald E (1999). Salt tolerance conferred by overexpression of a vacuolar Na+/H+ antiport in Arabidopsis. Science.

[MCU187C3] Azad A, Sawa Y, Ishikawa T, Shibata H (2004). Phosphorylation of plasma membrane aquaporin regulates temperature-dependent opening of tulip petals. Plant and Cell Physiology.

[MCU187C4] Azad A, Katsuhara M, Sawa Y, Ishikawa T, Shibata H (2008). Characterization of four plasma membrane aquaporins in tulip petals: a putative homolog is regulated by phosphorylation. Plant and Cell Physiology.

[MCU187C5] Barstow B, Ando N, Kim C, Gruner S (2008). Alteration of citrine structure by hydrostatic pressure explains the accompanying spectral shift. Proceedings of the National Academy of Sciences, USA.

[MCU187C6] Barstow B, Ando N, Kim C, Gruner S (2009). Coupling of pressure-induced structural shifts to spectral changes in a yellow fluorescent protein. Biophysical Journal.

[MCU187C7] Bassil E, Tajima H, Liang Y (2011). The Arabidopsis Na+/H+ antiporters NHX1 and NHX2 control vacuolar pH and K+ homeostasis to regulate growth, flower development, and reproduction. The Plant Cell.

[MCU187C8] Benitez-Alfonso Y, Jackson D, Maule A (2011). Redox regulation of intercellular transport. Protoplasma.

[MCU187C9] Benitez-Alfonso Y, Faulkner C, Pendle A, Miyashima S, Helariutta Y, Maule A (2013). Symplastic intercellular connectivity regulates lateral root patterning. Developmental Cell.

[MCU187C10] Benkert R, Obermeyer G, Bentrup F (1997). The turgor pressure of growing lily pollen tubes. Protoplasma.

[MCU187C11] van den Berg B, Wain R, Dobson C, Ellis R (2000). Macromolecular crowding perturbs protein refolding kinetics: implications for folding inside the cell. EMBO Journal.

[MCU187C12] Bieleski R, Elgar J, Heyes J (2000). Mechanical aspects of rapid flower opening in Asiatic lily. Annals of Botany.

[MCU187C13] Bots M, Feron R, Uehlein N, Weterings K, Kaldenhoff R, Mariani T (2005a). PIP1 and PIP2 aquaporins are differentially expressed during tobacco anther and stigma development. Journal of Experimental Botany.

[MCU187C14] Bots M, Vergeldt F, Wolters-Arts M, Weterings K, van As H, Mariani C (2005b). Aquaporins of the PIP2 class are required for efficient anther dehiscence in tobacco. Plant Physiology.

[MCU187C15] Boursiac Y, Boudet J, Postaire O, Luu D, Tournaire-Roux C, Maurel C (2008). Stimulus-induced downregulation of root water transport involves reactive oxygen species-activated cell signalling and plasma membrane intrinsic protein internalization. The Plant Journal.

[MCU187C16] Boyer J (1966). Isopiestic technique: measurement of accurate leaf water potentials. Science.

[MCU187C17] Boyer J (1995). Measuring the water status of plants and soils.

[MCU187C18] Boyer J, Knipling E (1965). Isopiestic technique for measuring leaf water potentials with a thermocouple psychrometer. Proceedings of the National Academy of Sciences, USA.

[MCU187C19] Briegel A, Ladinsky M, Oikonomou C (2014). Structure of bacterial cytoplasmic chemoreceptor arrays and implications for chemotactic signaling. eLife.

[MCU187C20] Burch-Smith T, Zambryski P (2012). Plasmodesmata paradigm shift: regulation from without versus within. Annual Review of Plant Biology.

[MCU187C21] Cazalé AC, Rouet-Mayer MA, Barbier-Brygoo H, Mathieu Y, Laurière C (1998). Oxidative burst and hypoosmotic stress in tobacco cell suspensions. Plant Physiology.

[MCU187C22] Chaumont F, Tyerman S (2014). Aquaporins: highly regulated channels controlling plant water relations. Plant Physiology.

[MCU187C23] Chaumont F, Moshelion M, Daniels M (2005). Regulation of plant aquaporin activity. Biology of the Cell.

[MCU187C24] Chebli Y, Geitmann A (2007). Mechanical principles governing pollen tube growth. Functional Plant Science and Biotechnology.

[MCU187C25] Chen W, Yin X, Wang L (2013). Involvement of rose aquaporin RhPIP1;1 in ethylene-regulated petal expansion through interaction with RhPIP2;1. Plant Molecular Biology.

[MCU187C26] Colombani M, Forterre Y (2011). Biomechanics of rapid movements in plants: poroelastic measurements at the cell scale. Computer Methods in Biomechanics and Biomedical Engineering.

[MCU187C27] Coster H (1976). Turgor pressure sensing in plant cell membranes. Plant Physiology.

[MCU187C28] Dashek W, Harwood H (1974). Proline, hydroxyproline, and lily pollen tube elongation. Annals of Botany.

[MCU187C158] Dainty J (1972). Plant cell-water relations: the elasticity of the cell wall. Proceedings of the Royal Society of Edinburgh. Section A. Mathematical and Physical Sciences.

[MCU187C29] Delauney A, Verma D (1993). Proline biosynthesis and osmoregulation in plants. The Plant Journal.

[MCU187C30] Deng Y, Sun M, Shaevitz J (2011). Direct measurement of cell wall stress stiffening and turgor pressure in live bacterial cells. Physical Review Letters.

[MCU187C31] van Doorn W, Van Meeteren U (2003). Flower opening and closure: a review. Journal of Experimental Botany.

[MCU187C32] Dumais J, Forterre Y (2012). Vegetable dynamicks’: the role of water in plant movements. Annual Review of Fluid Mechanics.

[MCU187C33] Ellis R (2001). Macromolecular crowding: an important but neglected aspect of the intracellular environment. Current opinion in structural biology.

[MCU187C34] Fetter K, Van Wilder V, Moshelion M, Chaumont F (2004). Interactions between plasma membrane aquaporins modulate their water channel activity. The Plant Cell.

[MCU187C159] Forouzesh E, Goel A, Mackenzie SA, Turner JA (2013). *In vivo* extraction of Arabidopsis cell turgor pressure using nanoindentation in conjunction with finite element modeling. The Plant Journal.

[MCU187C35] Forterre Y (2013). Slow, fast and furious: understanding the physics of plant movements. Journal of Experimental Botany.

[MCU187C36] Fricke W, Leigh R, Tomos A (1994). Concentrations of inorganic and organic solutes in extracts from individual epidermal, mesophyll and bundle-sheath cells of barley leaves. Planta.

[MCU187C37] Fricke W, Jarvis M, Brett C (2000). Turgor pressure, membrane tension and the control of exocytosis in higher plants. Plant, Cell and Environment.

[MCU187C38] Geitmann A (2006). Experimental approaches used to quantify physical parameters at cellular and subcellular levels. American Journal of Botany.

[MCU187C39] Geitmann A, Ortega J (2009). Mechanics and modeling of plant cell growth. Trends in Plant Science.

[MCU187C40] Gerbeau P, Amodeo G, Henzler T, Santoni V, Ripoche P, Maurel C (2002). The water permeability of Arabidopsis plasma membrane is regulated by divalent cations and pH. The Plant Journal.

[MCU187C41] Gerdenitsch W (1984). Microscopic contributions to the pressure–volume diagram of the cell-water relations as demonstrated with tissue cells. Protoplasma.

[MCU187C42] Gisel A, Barella S, Hempel F, Zambryski P (1999). Temporal and spatial regulation of symplastic trafficking during development in Arabidopsis thaliana apices. Development.

[MCU187C43] Green P (1968). Growth physics in Nitella: a method for continuous *in vivo* analysis of extensibility based on a micro-manometer technique for turgor pressure. Plant Physiology.

[MCU187C44] Griffiths A, Parry A, Jones H, Tomos A (1996). Abscisic acid and turgor pressure regulation in tomato roots. Journal of Plant Physiology.

[MCU187C45] Haines T (1994). Water transport across biological membranes. FEBS Letters.

[MCU187C46] Hamant O, Traas J (2010). The mechanics behind plant development. New Phytologist.

[MCU187C47] Han X, Hyun T, Zhang M (2014). Auxin–callose-mediated plasmodesmal gating is essential for tropic auxin gradient formation and signaling. Developmental Cell.

[MCU187C48] Hanson A, Kende H (1975). Ethylene-enhanced ion and sucrose efflux in morning glory flower tissue. Plant Physiology.

[MCU187C49] Harrington M, Razghandi K, Ditsch F (2011). Origami-like unfolding of hydro-actuated ice plant seed capsules. Nature Communications.

[MCU187C50] Haydon M, Bell L, Webb A (2011). Interactions between plant circadian clocks and solute transport. Journal of Experimental Botany.

[MCU187C51] Hayot C, Forouzesh E, Goel A, Avramova Z, Turner J (2012). Viscoelastic properties of cell walls of single living plant cells determined by dynamic nanoindentation. Journal of Experimental Botany.

[MCU187C52] Henzler T, Steudle E (2000). Transport and metabolic degradation of hydrogen peroxide in *Chara corallina*: model calculations and measurements with the pressure probe suggest transport of H_2_O_2_ across water channels. Journal of Experimental Botany.

[MCU187C53] Henzler T, Waterhouse R, Smyth A (1999). Diurnal variations in hydraulic conductivity and root pressure can be correlated with the expression of putative aquaporins in the roots of Lotus japonicus. Planta.

[MCU187C54] Hill A, Shachar-Hill B, Skepper J, Powell J, Shachar-Hill Y (2012). An osmotic model of the growing pollen tube. PloS One.

[MCU187C55] Holdaway-Clarke T, Walker N, Hepler P, Overall R (2000). Physiological elevations in cytoplasmic free calcium by cold or ion injection result in transient closure of higher plant plasmodesmata. Planta.

[MCU187C56] Hüsken D, Steudle E, Zimmermann U (1978). Pressure probe technique for measuring water relations of cells in higher-plants. Plant Physiology.

[MCU187C57] Imlau A, Truernit E, Sauer N (1999). Cell-to-cell and long-distance trafficking of the green fluorescent protein in the phloem and symplastic unloading of the protein into sink tissues. The Plant Cell.

[MCU187C58] Ishiguro S, Kawai-Oda A, Ueda J, Nishida I, Okada K (2001). The DEFECTIVE IN ANTHER DEHISCIENCE gene encodes a novel phospholipase A1 catalyzing the initial step of jasmonic acid biosynthesis, which synchronizes pollen maturation, anther dehiscence, and flower opening in Arabidopsis. The Plant Cell.

[MCU187C59] Javot H, Lauvergeat V, Santoni V (2003). Role of a single aquaporin isoform in root water uptake. The Plant Cell.

[MCU187C60] Johanson U, Karlsson M, Johansson I (2001). The complete set of genes encoding major intrinsic proteins in Arabidopsis provides a framework for a new nomenclature for major intrinsic proteins in plants. Plant Physiology.

[MCU187C61] Johnson K, Chrispeels M (1992). Tonoplast-bound protein kinase phosphorylates tonoplast intrinsic protein. Plant Physiology.

[MCU187C62] Joshi A, Knipfer T, Steudle E (2009). Effects of water storage in the stele on measurements of the hydraulics of young roots of corn and barley. New Phytologist.

[MCU187C63] Kamiya N, Tazawa M, Takata T (1963). The relation of turgor pressure to cell volume in nitella with special reference to mechanical properties of the cell wall. Protoplasma.

[MCU187C64] Keijzer C, Hoek I, Willemse M (1987). The processes of anther dehiscence and pollen dispersal III. The dehydration of the filament tip and the anther in three monocotyledonous species. New Phytologist.

[MCU187C65] Knipfer T, Das D, Steudle E (2007). During measurements of root hydraulics with pressure probes, the contribution of unstirred layers is minimized in the pressure relaxation mode: comparison with pressure clamp and high-pressure flowmeter. Plant, Cell and Environment.

[MCU187C66] Kochian LV, Shaff JE, Kühtreiber WM, Jaffe LF, Lucas WJ (1992). Use of an extracellular, ion-selective, vibrating microelectrode system for the quantification of K+, H+, and Ca2+ fluxes in maize roots and maize suspension cells. Planta.

[MCU187C67] Kolb K, Sperry J, Lamont B (1996). A method for measuring xylem hydraulic conductance and embolism in entire root and shoot systems. Journal of Experimental Botany.

[MCU187C68] Korolev A, Tomos A, Bowtell R, Farrar J (2000). Spatial and temporal distribution of solutes in the developing carrot taproot measured at single-cell resolution. Journal of Experimental Botany.

[MCU187C69] Kroeger J, Zerzour R, Geitmann A (2011). Regulator or driving force? The role of turgor pressure in oscillatory plant cell growth. PloS One.

[MCU187C70] Kühtreiber WM, Jaffe LF (1990). Detection of extracellular calcium gradients with a calcium-specific vibrating electrode. Journal of Cell Biology.

[MCU187C161] Kumar MN, Jane WN, Verslues PE (2013). Role of the putative osmosensor Arabidopsis Histidine Kinase1 in dehydration avoidance and low-water-potential response. Plant Physiology.

[MCU187C71] Lalonde S, Boles E, Hellmann H (1999). The dual function of sugar carriers. Transport and sugar sensing. The Plant Cell.

[MCU187C72] Lee S, Singh A, Chung G, Ahn S, Noh E, Steudle E (2004). Exposure of roots of cucumber (Cucumis sativus) to low temperature severely reduces root pressure, hydraulic conductivity and active transport of nutrients. Physiologia Plantarum.

[MCU187C73] Lei B, Huang Y, Sun J (2014). Scanning ion-selective electrode technique and X-ray microanalysis provide direct evidence of contrasting Na+ transport ability from root to shoot in salt-sensitive cucumber and salt-tolerant pumpkin under NaCl stress.

[MCU187C74] Li W, Zhao Y, Liu C (2012). Callose deposition at plasmodesmata is a critical factor in restricting the cell-to-cell movement of Soybean mosaic virus. Plant Cell Reports.

[MCU187C75] Liarzi O, Epel B (2005). Development of a quantitative tool for measuring changes in the coefficient of conductivity of plasmodesmata induced by developmental, biotic, and abiotic signals. Protoplasma.

[MCU187C76] Lintilhac P, Wei C, Tanguay J, Outwater J (2000). Ball tonometry: a rapid, nondestructive method for measuring cell turgor pressure in thin-walled plant cells. Journal of Plant Growth Regulation.

[MCU187C77] Lockhart JA (1965). An analysis of irreversible plant cell elongation. Journal of Thoeretical Biology.

[MCU187C78] Lucas M, Kenobi K, von Wangenheim D (2013). Lateral root morphogenesis is dependent on the mechanical properties of the overlaying tissues. Proceedings of the National Academy of Sciences, USA.

[MCU187C79] Lucas W, Lee J (2004). Plasmodesmata as a supracellular control network in plants. Nature Reviews Molecular Cell Biology.

[MCU187C80] Ma N, Xue J, Li Y (2008). Rh-PIP2;1, a rose aquaporin gene, is involved in ethylene-regulated petal expansion. Plant Physiology.

[MCU187C81] Martre P, Morillon R, Barrieu F, North G, Nobel P, Chrispeels M (2002). Plasma membrane aquaporins play a significant role during recovery from water deficit. Plant Physiology.

[MCU187C160] Matsui T, Omasa K, Horie T (2000). Anther dehiscence in two-rowed barley (*Hordeum distichum*) triggered by mechanical stimulation. Journal of Experimental Botany.

[MCU187C82] Mattioli R, Falasca G, Sabatini S, Altamura M, Costantino P, Trovato M (2009). The proline biosynthetic genes P5CS1 and P5CS2 play overlapping roles in Arabidopsis flower transition but not in embryo development. Physiologia Plantarum.

[MCU187C83] Maurel C, Verdoucq L, Luu D, Santoni V (2008). Plant aquaporins: membrane channels with multiple integrated functions. Annual Review of Plant Biology.

[MCU187C84] Meshcheryakov A, Steudle E, Komor E (1992). Gradients of turgor, osmotic pressure, and water potential in the cortex of the hypocotyl of growing ricinus seedlings: effects of the supply of water from the xylem and of solutes from the Phloem. Plant Physiology.

[MCU187C85] Messerli MA, Smith PJS, Lewis RC, Robinson KR (2004). Chloride fluxes in lily pollen tubes: a critical reevaluation. The Plant Journal.

[MCU187C86] Milani P, Braybrook S, Boudaoud A (2013). Shrinking the hammer: micromechanical approaches to morphogenesis. Journal of Experimental Botany.

[MCU187C87] Molz FJ, Boyer JS (1978). Growth-induced water potentials in plant cells and tissues. Plant Physiology.

[MCU187C88] Monshausen G, Haswell E (2013). A force of nature: molecular mechanisms of mechanoperception in plants. Journal of Experimental Botany.

[MCU187C89] Morgan J (1984). Osmoregulation and water stress in higher plants. Annual Review of Plant Physiology.

[MCU187C90] Munns R, Greenway H, Setter T, Kuo J (1983). Turgor pressure, volumetric elastic modulus, osmotic volume and ultrastructure of *Chlorella emersonii* grown at high and low external NaCl. Journal of Experimental Botany.

[MCU187C91] Murata K, Mitsuoka K, Hirai T (2000). Structural determinants of water permeation through aquaporin-1. Nature.

[MCU187C92] Nelson M, Band L, Dyson R (2012). A biomechanical model of anther opening reveals the roles of dehydration and secondary thickening. New Phytologist.

[MCU187C93] Niemietz C, Tyerman S (2002). New potent inhibitors of aquaporins: silver and gold compounds inhibit aquaporins of plant and human origin. FEBS Letters.

[MCU187C94] Nonami H, Boyer J, Steudle E (1987). Pressure probe and isopiestic psychrometer measure similar turgor. Plant Physiology.

[MCU187C95] Oparka K, Prior D (1992). Direct evidence for pressure-generated closure of plasmodesmata. The Plant Journal.

[MCU187C96] Oparka K, Prior D, Wright K (1995). Symplastic communication between primary and developing lateral roots of *Arabidopsis thaliana*. Journal of Experimental Botany.

[MCU187C97] Parre E, Geitmann A (2004). More than a leak sealant. The mechanical properties of callose in pollen tubes. Plant Physiology.

[MCU187C98] Pei H, Ma N, Tian J (2013). An NAC transcription factor controls ethylene-regulated cell expansion in flower petals. Plant Physiology.

[MCU187C99] Perbal M, Haughn G, Saedler H, Schwarz-Sommer Z (1996). Non-cell-autonomous function of the Antirrhinum floral homeotic proteins DEFICIENS and GLOBOSA is exerted by their polar cell-to-cell trafficking. Development.

[MCU187C100] Péret B, Li G, Zhao J (2012). Auxin regulates aquaporin function to facilitate lateral root emergence. Nature Cell Biology.

[MCU187C101] Peters W, Tomos A (1996). The history of tissue tension. Annals of Botany.

[MCU187C102] Postaire O, Tournaire-Roux C, Grondin A (2010). A PIP1 aquaporin contributes to hydrostatic pressure-induced water transport in both the root and rosette of Arabidopsis. Plant Physiology.

[MCU187C103] Prado K, Maurel C (2013). Regulation of leaf hydraulics: from molecular to whole plant levels. Frontiers in Plant Science.

[MCU187C104] Prado K, Boursiac Y, Tournaire-Roux C (2013). Regulation of Arabidopsis leaf hydraulics involves light-dependent phosphorylation of aquaporins in veins. The Plant Cell.

[MCU187C105] Ramahaleo T, Morillon R, Alexandre J, Lassalles J (1999). Osmotic water permeability of isolated protoplasts. Modifications during development. Plant Physiology.

[MCU187C106] Rehman S, Yun S (2006). Developmental regulation of K accumulation in pollen, anthers, and papillae: are anther dehiscence, papillae hydration, and pollen swelling leading to pollination and fertilization in barley (Hordeum vulgare L.) regulated by changes in K concentration?. Journal of Experimental Botany.

[MCU187C107] Reid M, Evans R, Dodge L, Mor Y (1989). Ethylene and silver thiosulfate influence opening of cut rose flowers. Journal of the American Society for Horticultural Science.

[MCU187C108] Riesmeier J, Willmitzer L, Frommer W (1994). Evidence for an essential role of the sucrose transporter in phloem loading and assimilate partitioning. EMBO Journal.

[MCU187C109] Rinne P, van der Schoot C (1998). Symplasmic fields in the tunica of the shoot apical meristem coordinate morphogenetic events. Development.

[MCU187C110] Robinson S, Burian A, Couturier E (2013). Mechanical control of morphogenesis at the shoot apex. Journal of Experimental Botany.

[MCU187C111] Routier-Kierzkowska A, Smith R (2012). Measuring the mechanics of morphogenesis. Current Opinion in Plant Biology.

[MCU187C112] Routier-Kierzkowska A, Weber A, Kochova P (2012). Cellular force microscopy for *in vivo* measurements of plant tissue mechanics. Plant Physiology.

[MCU187C113] Ruan Y, Llewellyn D, Furbank R (2001). The control of single-celled cotton fiber elongation by developmentally reversible gating of plasmodesmata and coordinated expression of sucrose and K+ transporters and expansin. The Plant Cell.

[MCU187C114] Rutschow H, Baskin T, Kramer E (2011). Regulation of solute flux through plasmodesmata in the root meristem. Plant Physiology.

[MCU187C115] Rygol J, Pritchard J, Zhu J, Tomos A, Zimmermann U (1993). Transpiration induces radial turgor pressure gradients in wheat and maize roots. Plant Physiology.

[MCU187C116] Sack L, Holbrook N (2006). Leaf hydraulics. Annual Review of Plant Biology.

[MCU187C117] Sack L, Melcher PJ, Zwieniecki MA, Holbrook NM (2002). The hydraulic conductance of the angiosperm leaf lamina: a comparison of three measurement methods. Journal of Experimental Botany.

[MCU187C118] Sanati Nezhad A, Geitmann A (2013). The cellular mechanics of an invasive lifestyle. Journal of Experimental Botany.

[MCU187C119] Scholander P, Hammel H, Hemmingsen E, Bradstreet E (1964). Hydrostatic pressure and osmotic potential in leaves of mangroves and some other plants. Proceedings of the National Academy of Sciences, USA.

[MCU187C120] Schwacke R, Grallath S, Breitkreuz K (1999). LeProT1, a transporter for proline, glycine betaine, and gamma-amino butyric acid in tomato pollen. The Plant Cell.

[MCU187C121] Scott R, Spielman M, Dickinson H (2004). Stamen structure and function. The Plant Cell.

[MCU187C122] Sessions A, Yanofsky M, Weigel D (2000). Cell–cell signaling and movement by the floral transcription factors LEAFY and APETALA1. Science.

[MCU187C123] Shabala S, Lew R (2002). Turgor regulation in osmotically stressed Arabidopsis epidermal root cells. Direct support for the role of inorganic ion uptake as revealed by concurrent flux and cell turgor measurements. Plant Physiology.

[MCU187C124] Shachar-Hill B, Hill A, Powell J, Skepper J, Shachar-Hill Y (2013). Mercury-sensitive water channels as possible sensors of water potentials in pollen. Journal of Experimental Botany.

[MCU187C125] Sibaoka T (1969). Physiology of rapid movements in higher plants. Annual Review of Plant Physiology.

[MCU187C126] Sivitz A, Reinders A, Ward J (2008). Arabidopsis sucrose transporter AtSUC1 is important for pollen germination and sucrose-induced anthocyanin accumulation. Plant Physiology.

[MCU187C127] Stadler R, Truernit E, Gahrtz M, Sauer N (1999). The AtSUC1 sucrose carrier may represent the osmotic driving force for anther dehiscence and pollen tube growth in Arabidopsis. The Plant Journal.

[MCU187C128] Steudle E, Oren R, Schulze ED (1987). Measurement of hydraulic conductivity, solute permeability, and of reflection coefficients of excised roots using the root pressure probe. Plant Physiology.

[MCU187C129] Szabados L, Savouré A (2010). Proline: a multifunctional amino acid. Trends in Plant Science.

[MCU187C130] Taiz L, Zeiger E (2010). Plant physiology.

[MCU187C131] Talbott L, Zeiger E (1998). The role of sucrose in guard cell osmoregulation. Journal of Experimental Botany.

[MCU187C132] Tang W, He Y, Tu L (2014). Down-regulating annexin gene GhAnn2 inhibits cotton fiber elongation and decreases Ca2+ influx at the cell apex. Plant Molecular Biology.

[MCU187C133] Tomos A, Leigh R (1999). The pressure probe: a versatile tool in plant cell physiology. Annual Review of Plant Physiology and Plant Molecular Biology.

[MCU187C134] Tournaire-Roux C, Sutka M, Javot H (2003). Cytosolic pH regulates root water transport during anoxic stress through gating of aquaporins. Nature.

[MCU187C135] Trolinder N, Mcmichael B, Upchurch D (1993). Water relations of cotton flower petals and fruit. Plant, Cell and Environment.

[MCU187C136] Tsuda M, Tyree M (2000). Plant hydraulic conductance measured by the high pressure flow meter in crop plants. Journal of Experimental Botany.

[MCU187C137] Tyree M, Hammel H (1972). The measurement of the turgor pressure and the water relations of plants by the pressure-bomb technique. Journal of Experimental Botany.

[MCU187C138] Tyree M, Patiño S, Bennink J, Alexander J (1995). Dynamic measurements of root hydraulic conductance using a high-pressure flowmeter in the laboratory and field. Journal of Experimental Botany.

[MCU187C139] Tyree M, Nardini A, Salleo S, Sack L, El Omari B (2005). The dependence of leaf hydraulic conductance on irradiance during HPFM measurements: any role for stomatal response?. Journal of Experimental Botany.

[MCU187C140] Urao T, Yakubov B, Satoh R (1999). A transmembrane hybrid-type histidine kinase in Arabidopsis functions as an osmosensor. The Plant Cell.

[MCU187C141] Vatén A, Dettmer J, Wu S (2011). Callose biosynthesis regulates symplastic trafficking during root development. Developmental Cell.

[MCU187C162] Vella D, Ajdari A, Vaziri A, Boudaoud A (2012a). Indentation of ellipsoidal and cylindrical elastic shells. Physical Review Letters.

[MCU187C142] Vella D, Ajdari A, Vaziri A, Boudaoud A (2012b). The indentation of pressurized elastic shells: from polymeric capsules to yeast cells. Journal of the Royal Society, Interface/the Royal Society.

[MCU187C143] Verbruggen N, Hermans C (2008). Proline accumulation in plants: a review. Amino Acids.

[MCU187C144] Vermeer J, von Wangenheim D, Barberon M (2014). A spatial accommodation by neighboring cells is required for organ initiation in Arabidopsis. Science.

[MCU187C145] Vogler H, Draeger C, Weber A (2012). The pollen tube: a soft shell with a hard core. The Plant Journal.

[MCU187C146] Walley JW, Coughlan S, Hudson ME (2007). Mechanical stress induces biotic and abiotic stress responses via a novel cis-element. PLoS Genetics.

[MCU187C147] Wang L, Hukin D, Pritchard J, Thomas C (2006). Comparison of plant cell turgor pressure measurement by pressure probe and micromanipulation. Biotechnology Letters.

[MCU187C148] Watanabe T, Imada K, Yoshizawa K (2013). Glycine insertion makes yellow fluorescent protein sensitive to hydrostatic pressure. PLoS One.

[MCU187C149] Wendler S, Zimmermann U (1982). A new method for the determination of hydraulic conductivity and cell volume of plant cells by pressure clamp. Plant Physiology.

[MCU187C150] Wolf S, Deom C, Beachy R, Lucas W (1989). Movement protein of tobacco mosaic virus modifies plasmodesmatal size exclusion limit. Science.

[MCU187C151] Yang C, Xu Z, Song J (2007). Arabidopsis MYB26/MALE STERILE35 regulates secondary thickening in the endothecium and is essential for anther dehiscence. The Plant Cell.

[MCU187C152] Yooyongwech S, Horigane A, Yoshida M (2008). Changes in aquaporin gene expression and magnetic resonance imaging of water status in peach tree flower buds during dormancy. Physiologia Plantarum.

[MCU187C153] Zhang WH, Tyerman SD (1999). Inhibition of water channels by HgCl_2_ in intact wheat root cells. Plant Physiolgy.

[MCU187C154] Zimmermann U (1978). Physics of turgor and osmoregulation. Annual Review of Plant Physiology.

[MCU187C155] Zimmermann U, Hüsken D (1979). Theoretical and experimental exclusion of errors in the determination of the elasticity and water transport parameters of plant cells by the pressure probe technique. Plant Physiology.

[MCU187C156] Zimmermann U, Steudle E, Lelkes P (1976). Turgor pressure regulation in Valonia utricularis: effect of cell wall elasticity and auxin. Plant Physiology.

[MCU187C157] Zimmermann U, Rygol J, Balling A, Klöck G, Metzler A, Haase A (1992). Radial turgor and osmotic pressure profiles in intact and excised roots of Aster tripolium: pressure probe measurements and nuclear magnetic resonance-imaging analysis. Plant Physiology.

